# Electroacupuncture alleviates blood-brain barrier disruption and neuroinflammation via astrocytic MC4R in a mouse model of multiple sclerosis

**DOI:** 10.1186/s12974-025-03667-1

**Published:** 2025-12-26

**Authors:** Yanping Wang, Xiaoru Ma, Zhixin Qiao, Xiyu Zhang, Jiayu Ji, Sifan Zhang, Wei Zhuang, Junfeng Wu, Anqi Li, Chao Wang, Xin Xiu, Jing Wang, Yanting Meng, Wei Huang, Xiujuan Lang, Xijun Liu, Bo Sun, Hulun Li, Yumei Liu

**Affiliations:** 1 Department of Neurobiology, School of Basic Medical Sciences, Harbin Medical University, Harbin, 150081 Heilongjiang PR China; 2 The Key Laboratory of Preservation of Human Genetic Resources and Disease Control in China, Harbin Medical University, Ministry of Education, Harbin, 150081 Heilongjiang PR China; 3 The Key Laboratory of Myocardial Ischemia, Harbin Medical University, Ministry of Education, Harbin, 150081 Heilongjiang PR China

**Keywords:** Melanocortin-4 receptors (MC4R), Α-melanocyte-stimulating hormone (α-MSH), Experimental autoimmune encephalomyelitis (EAE), Blood-brain barrier (BBB), Electroacupuncture (EA)

## Abstract

Astrocytes are key regulators of neuroinflammation in multiple sclerosis (MS). Electroacupuncture (EA), a safe and cost-effective adjuvant therapy, has shown benefits in neurodegenerative diseases, but its astrocyte-related mechanisms remain unclear. Here, we demonstrated that EA at ST36 alleviated blood-brain barrier (BBB) disruption and neuroinflammation during the peak period of experimental autoimmune encephalomyelitis (EAE). Additionally, EA at ST36 upregulated the expression of α-melanocyte-stimulating hormone (α-MSH) and its receptor melanocortin-4 receptor (MC4R) in spinal astrocytes. Pharmacological studies showed that MC4R agonist RO27-3225 mimicked the therapeutic effects of EA, whereas MC4R antagonist TCMCB07 weakened EA-mediated BBB protection and neuroinflammation suppression. Moreover, astrocyte-specific silencing of MC4R via adeno-associated virus (AAV) weakened EA-mediated BBB protection and neuroinflammation suppression. RNA-sequencing (RNA-seq) and western blot (WB) revealed that EA exerts neuroprotective effects by activating MC4R to inhibit MAPK and NF-κB signaling pathways. Moreover, in MC4R-overexpressing astrocytes, α-MSH and RO27-3225 reduced inflammation responses, while TCMCB07 reversed the effects by MAPK/NF-κB signaling pathways. Collectively, our findings identify astrocytic MC4R as a critical mediator of EA-driven neuroprotection by suppressing MAPK/NF-κB signaling, providing mechanistic insight and a promising therapeutic target for EAE and other neuroinflammatory disorders.

**Supplementary Information:**

The online version contains supplementary material available at 10.1186/s12974-025-03667-1.

## Introduction

Multiple sclerosis (MS) is a chronic inflammatory and neurodegenerative disease and remains the leading cause of non-traumatic disability in young adults worldwide [1, 2]. Its pathological hallmarks include persistent neuroinflammation, blood-brain barrier (BBB) breakdown, demyelination and axonal loss, reflecting widespread central nervous system (CNS) injury [3]. Increasing evidences have identified astrocytes as key amplifiers of autoimmune inflammation in MS. Upon activation, astrocytes release substantial amounts of inflammatory factors and neurotoxic substances, trigger neuroinflammation and compromise BBB integrity [4–8].

Electroacupuncture (EA) at Zusanli (ST36) has been widely investigated for its immunomodulatory effects in inflammatory and neurological disorders [9–12]. In the context of experimental autoimmune encephalomyelitis (EAE), EA at ST36 has been shown to suppress pro-inflammatory pathways, including miR-155-mediated signaling [13], regulate T-cell activation and differentiation [14], promote remyelination by modulating stem cell proliferation and oligodendrocyte lineage commitment [15, 16]. Although these findings highlight multiple downstream mechanisms of EA, emerging neuroanatomical and hormonal evidences suggest that these diverse effects may converge on a common upstream-hypothalamic pro-opiomelanocortin (POMC) neurons. EA has been demonstrated to activate POMC neurons and increase the production of α-melanocyte-stimulating hormone (α-MSH), a neuropeptide derived from the precursor protein POMC [17–19] and with potent anti-inflammatory and neuroprotective actions [20].

α-MSH exerts its biological actions through five melanocortin receptors (MC1R-MC5R) [21]. Previous works have revealed a protective role of MC1R activation in EAE and other inflammatory CNS conditions [22, 23]. However, it is also reported that MC1R activation in the CNS is not involved in the anti-inflammatory actions of α-MSH [24]. Moreover, accumulating data indicate that MC4R rather than MC1R is the most widely expressed melanocortin receptor in the CNS and is particularly enriched in astrocytes [25, 26]. Previous work has revealed that MC4R not only regulates energy balance in the hypothalamus but also played a critical role in neuroinflammation [27]. The effect of neuroprotection and anti-inflammatory actions of MC4R has been studied in intracerebral hemorrhage damage [28], cerebral ischemia damage [29] and spinal cord injury [30, 31]. MC4R signaling in astrocytes has been shown to exert anti-inflammatory actions and promote an anti-inflammatory macrophage phenotype [32–34]. Especially, MC4R expression is markedly upregulated in astrocytes within MS lesions [34], suggesting a potential compensatory or regulatory role during autoimmune neuroinflammation. Despite these advances, the contribution of astrocytic MC4R to EAE pathology remains largely unexplored. It remains still unclear whether the therapeutic benefit of EA in EAE are mediated through astrocytic MC4R signaling and what the underlying mechanism entails. Therefore, in this study, we hypothesized that EA engages the α-MSH-MC4R signaling to restrain astrocyte-mediated inflammatory responses, thereby contributing to functional recovery in EAE.

## Materials and methods

### Animals

In this experiment, female C57BL/6J mice aged 6–8 weeks were purchased from HFK Bioscience Co., Ltd (Beijing, China), and housed in a special pathogen-free (SPF) animal facility with 12 h light and dark circulation, temperature of 22–24℃ and humidity of 55 ± 5%. Before the experiment, all mice were allowed to adapt to the environment for at least one week with free access to food.

### EAE

EAE was established using rMOG (200 µg/animal) emulsified in complete freund’s adjuvant (CFA) (Sigma, USA) containing 5 mg/mL *mycobacterium tuberculosis* (BD Biosciences, USA) and i.v. injected with pertussis toxin (300 ng/animal; List Biological Laboratories, USA) on day 0 and 2. The mice were examined for disease signs and scored on a scale of 0–5: 0, no clinical signs; 1, paralyzed tail; 2, paralysis; 3, paraplegia; 4, paraplegia with forelimb weakness or paralysis; 5, near-death state or death, between the two scoring standards to give plus or minus 0.5 points.

### In vivo experimental grouping

The study consisted of three experimental parts. EA treatment: mice were randomly assigned to the CFA control group (CFA), EAE group (EAE) and EAE + EA at ST36 group (EAE + EA). In EAE + EA group, EA at ST36 was started from day 1 post-immunization. ST36 was located 3 mm below the knee joint in the muscle sulcus, with a needle depth of 2 mm, and EA was performed for 30 min daily. Pharmacological intervention: mice were divided into the following groups: EAE + saline group (EAE + Saline), EAE + EA at ST36 + saline group (EAE + EA + Saline), EAE + RO27-3225 group (EAE + RO27-3225) and EAE + EA at ST36 + TCMCB07 group (EAE + EA + TCMCB07). RO27-3225, TCMCB07 and saline was i.v. administered every other day, 30 min prior to EA. AAV-mediated MC4R silencing: MC4R expression in astrocytes was silenced by intrathecal injection of adeno-associated virus (AAV), and EAE were induced after 28 days. The experiment was divided into four groups: AAV-GFAP-shNC + EAE group, AAV-GFAP-shNC + EAE + EA group, AAV-GFAP-shMC4R + EAE group, and AAV-GFAP-shMC4R + EAE + EA group.

### The treatment of agonists and antagonist of MC4R

To investigate the role of MC4R in EA at ST36 in the treatment of EAE, we selected the specific agonist RO27-3225 (150 µg/kg) and the specific antagonist TCMCB07 (250 µg/kg) of MC4R to activate or inhibit MC4R through i.v. injection every other day 30 min before EA. Normal saline was injected in the same manner as a control.

### Intrathecal injection of AAV

To selectively silence MC4R expression in astrocytes, AAV5 vectors (Genechem, Shanghai, China) carrying shRNAmiR sequences under the control of the GFAP promoter and EGFP reporter were constructed. Three shRNA constructs targeting different sequences of MC4R were designed: AAV5-GFAP-shMC4R-(P24K1247): CCATCGTCATTACCCTGTTAA; AAV5-GFAP-shMC4R-(P24K1248): CGTCATCGACCCTCTCATTTA; AAV5-GFAP-shMC4R-(P24K1249): AGAATCCATACTGCGTGTGCT), and a negative control (AAV5-GFAP-shNC: TTCTCCGAACGTGTCACGT). For intrathecal injection, mice were anesthetized with tribromoethanol, and 10 µL of viral stock solution (1 × 10^11^ vg/mL) was injected between the L5-L6 intervertebral space. A sudden slight upward flick of the tail indicated successful intrathecal insertion.

### Isolation of primary mouse astrocytes

Brain tissues from 1-3-day-old neonatal mice were dissected to isolate the cortex and hippocampus. The tissue fragments were gently triturated repeatedly until a single-cell suspension obtained. After centrifugation, the cell pellet was resuspended in DMEM/F-12 complete medium supplemented with 10% fetal bovine serum (FBS) (YEASEN, China) and 1% penicillin-streptomycin (Thermo Fisher Scientific, USA). The cells were cultured at 37 °C and 5% CO2, and the complete medium was changed every 3 days. After one week, the culture flasks were transferred to a shaker to purify astrocytes, and purified astrocytes were used for experiments.

### Adenovirus transfection of primary astrocytes

The primary astrocyte adenovirus transfection reagent was designed and synthesized by Genechem (Shanghai, China). The transfection methods were carried out according to the manufacturer’s instructions: to confirm the appropriate infection conditions, the experiment was divided into two groups: culture medium (CM) group and Enhanced Infection Solution (EIS) group. Primary astrocytes at 50% confluence were prepared. The adenovirus was serially diluted in either CM or EIS to different Multiplicity of Infection (MOI) values: 10,000, 1,000, 100, 10, and 1. The infection status was analyzed after 48–72 h. Primary astrocytes transfected with adenovirus expressed EGFP.

### Cell cultures

To assess lipopolysaccharide (LPS) (Sigma, USA)-induced inflammatory injury in astrocytes at different time points, primary astrocytes at 80% confluence were treated with LPS and subsequently collected at designated time points (0, 0.5, 1, 2, 4, 8, 12, 24 h). To investigate the effect of MC4R on LPS-induced inflammatory injury in astrocytes, primary astrocytes at 80% confluence were pretreated with complete medium containing α-MSH (1 µM), RO27-3225 (1 µM), or TCMCB07 (1 µM) for 1 h, followed by LPS stimulation for either 1–4 h before cell collection. To elucidate the regulatory effect of α-MSH on MAPK and NF-κB signaling pathways, primary astrocytes overexpressing MC4R at 80% confluence were pretreated for 30 min with complete medium containing U0126 (10 µM, an ERK pathway inhibitor), SP600125 (10 µM, a JNK pathway inhibitor), SB203580 (10 µM, a p38 MAPK pathway inhibitor) or NF-κB-IN-11(10 µM, an NF-κB pathway inhibitor). Subsequently, cells were pretreated for 30 min with α-MSH (1 µM), followed by LPS stimulation for 4 h before cell collection. The reagents used were listed in Supplementary Table 5.

### RNA isolation and quantitative real-time PCR (qPCR)

Total RNA was extracted from tissues or cells using Trizol and the concentration of total RNA was measured by using Nanodrop 2000 (Thermo Fisher Scientific, USA). cDNA was synthesized using Reverse Transcriptase M-MLV (TaKaRa, Japan) and dNTPs (TaKaRa, Japan). Subsequently, qPCR analysis was performed using StepOne Plus fluorescent quantitative PCR instrument (ABI, USA). Relative gene expression levels of mRNA were analyzed using the ΔΔCT method. The primer sequences used were listed in Supplementary Table 1.

### RNA-sequencing (RNA-seq)

Total RNA was extracted from spinal cord tissues and submitted to the Genomics Institute (Beijing, China) for library construction and sequencing on the DNBSEQ platform (paired-end 150 bp). Hisat2 and Bowtie2 were used to evaluate the quality of RNA-seq results. The gene expression levels were quantified with RSEM software, with the results were normalized and reported as TPM/FPKM. Differentially expressed genes (DEGs) were identified using DESeq2, with significance defined as false discovery rate (FDR) ≤ 0.001 or Q value ≤ 0.05. Gene Ontology (GO) and Kyoto Encyclopedia of Genes and Genomes (KEGG) pathway enrichment analyses were performed using the hypergeometric test, with Q value ≤ 0.05 considered significant.

### Hematoxylin and Eosin (H&E) and FluoroMyelin^™^ green staining

Mice were anesthetized with 2% sodium pentobarbital, followed by cardiac perfusion with phosphate-buffered saline (PBS). The spinal cord was then placed in 4% paraformaldehyde (PFA) at 4 °C overnight, and dehydrated with 30% sucrose solution. After that, the lumbar spinal cord sections were prepared at 10 μm thickness using a cryostat (Thermo Fisher Scientific, USA). H&E (Solarbio, China) and FluoroMyelin™ green staining (Invitrogen, USA) were performed to detect inflammatory cell infiltration and demyelination in the spinal cord, respectively.

### Immunofluorescence staining

Cells or tissue sections were fixed, permeabilized, and incubated with primary antibodies overnight at 4°C, followed by incubation with secondary antibody and counterstained with 4’,6-diamidino-2-phenylindole (DAPI). Fluorescence images were captured using confocal microscope (LSM 700; Carl Zeiss, Germany) and quantified using Image J (NIH, USA). We applied Subtract Background function to uniformly suppress uneven background fluorescence across all samples. A consistent threshold was used to distinguish true signal from residual background. No manual adjustments were made between samples. Region of interests (ROI) were selected from analysis. The antibodies used were listed in Supplementary Table 2.

### Transmission electron microscopic (TEM)

The brain and spinal cord were obtained and fixed separately in 2.5% glutaraldehyde for 2 h, followed by 1% osmium tetroxide for 2 h, dehydrated and embedded in epoxy resin. Section stained with toluidine blue or uranium acetate and lead citrate, then observed by TEM (Hitachi, Japan). Each myelin sheath was tracked using the ImageJ plugin G-ratio calculator (G-ratio = axon diameter/total diameter of myelinated fiber).

### Evans Blue (EB) extravasation test

4% EB (4 ml/kg) (Sigma, USA) was i.v. administered and the mice were anesthetized and perfused with PBS 2 h later. The entire brain and spinal cord were removed, weighed and homogenized in PBS, respectively. The homogenate supernatants were mixed with an equal volume of 50% trichloroacetic acid, incubated overnight at 4 °C, and centrifuged at 12,000 × g for 15 min. The supernatant was collected and measured at 620 nm with Microplate reader (Tecan, Switzerland).

### Preparation of brain mononuclear cell

The brain tissues were homogenized by a grinder, filtered, centrifuged, and digested with 1640 medium containing 0.1% collagenase (Sigma, USA) for 1 h. The pellet was collected by centrifugation, resuspended in 30% percoll (Cytiva, UK) and centrifuged at 400 × g for 30 min at 20 °C (no break). Then mononuclear cells in the lower precipitate were collected.

### Flow Cytometry (FCM)

For surface marker staining, single-cell suspensions were first incubated with fixable viability dye eFluor™ 780 (FVD; BioLegend, USA) for 30 min in the dark, followed by surface antibody staining for 30 min at 4 °C. For intracellular cytokine detection, cells were stimulated for 4 h at 37 °C in complete medium containing 50 ng/mL PMA (Sigma, USA), 1 µg/mL ionomycin (Sigma, USA), and 1× brefeldin A (BioLegend, USA). After stimulation, cells were stained with FVD and surface antibodies as described above, fixed and permeabilized using the BD Cytofix/Cytoperm kit (BD Biosciences, USA), and then incubated with intracellular antibodies for 30 min at 4 °C. Cells were then fixed with 1% PFA. Data were acquired on a FACSVerse flow cytometer (BD Biosciences, USA) and analyzed using FlowJo software (Version X, USA). The antibodies used were listed in Supplementary Table 3.

### Western Blot (WB)

Tissues or cells were homogenized in RIPA lysis buffer (Beyotime, China) supplemented with 1% PMSF (Beyotime, China) and 1% phosphatase inhibitor (Biosharp, China). The lysate concentration was detected by BCA protein quantitative kit (Abbkine, China). The lysates were separated, transferred, and incubated with primary antibody overnight at 4 °C and then secondary antibody labeled with horseradish peroxidase for 1 h at room temperature. The protein bands were acquired using Amersham Imager 600 instrument (GE, USA), and analyzed with ImageJ software. The antibodies used were listed in Supplementary Table 4.

### Enzyme Linked Immunosorbent Assay (ELISA)

The spinal cord tissues were homogenized in RIPA lysis buffer supplemented with 1% PMSF and 1% phosphatase inhibitor. The lysates were subjected to the Mouse alpha MSH Elisa kit (BlueGene, China) according to the manufacturer’s instructions and analyzed at 450 nm.

### Statistical analysis

All experimental data were analyzed by GraphPad Prism 10 and Image J. Data were analyzed using an unpaired *t*-test between two groups or one-way or two-way ANOVA between more than two independent groups. All the data were expressed as the Means ± SD, with statistical significance (*P < 0.05; **P < 0.01; ***P < 0.001).

## Results

### EA at ST36 upregulated the expression of α-MSH and MC4R in the spinal cord of EAE

**Fig. 1:**
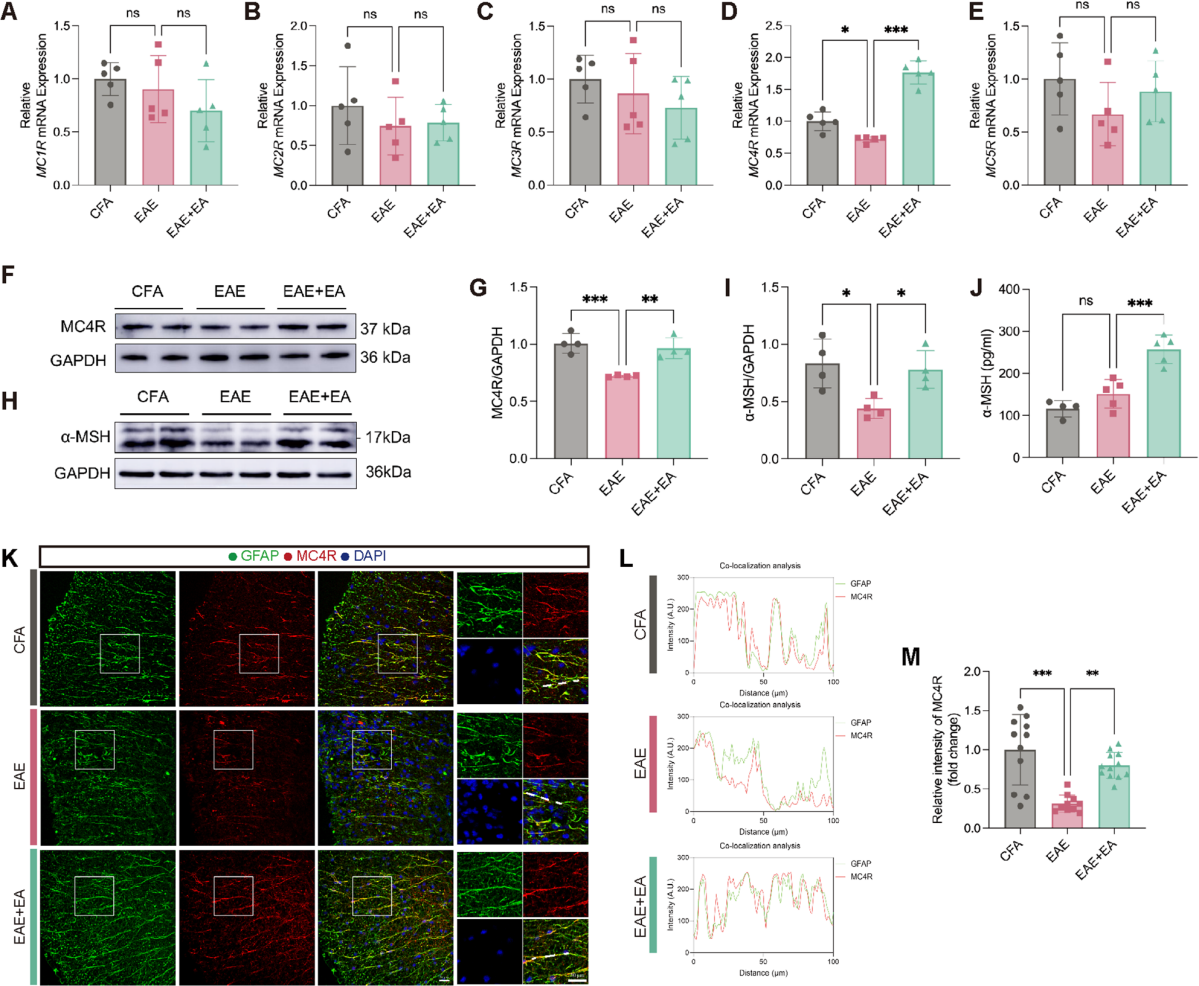
EA at ST36 upregulated the expression of α-MSH and MC4R in the spinal cord of EAE. (**A-E**) qPCR analysis of *MC1R-MC5R*. *n* = 5. (**F**) WB analysis of MC4R. (**G**) The statistical results of MC4R in F. *n* = 4. (**H**) WB analysis of α-MSH. (**I**) The statistical results of α-MSH in H. *n* = 4. (**J**) ELISA analysis of α-MSH. *n* ≥ 4. (**K**) Representative images of MC4R and GFAP of immunofluorescence staining. Scale bars: 50 μm. (**L**) The co-localization analysis of MC4R and GFAP. (**M**) The statistical results of MC4R intensity in K. *n* = 5. One-way ANOVA is used in A-E, G, I-J, M

To investigate the expression of melanocortin receptors in the spinal cord, we measured the mRNA levels of *MC1R*-*MC5R* by qPCR on day 15 post-immunization. Among these receptors, only *MC4R* expression was significantly decreased in the EAE group compared with the CFA group, whereas EA treatment markedly restored its expression. No significant changes were observed in *MC1R*, *MC2R*, *MC3R*, or *MC5R* (Fig. 1A-E). WB analysis further confirmed the downregulation of MC4R in EAE and its restoration by EA (Fig. 1F-G). Moreover, both WB and ELISA indicated that EA at ST36 significantly upregulated α-MSH levels in the spinal cord (Fig. 1H-J). To determine the cellular localization of MC4R, we performed immunofluorescence staining. MC4R was exclusively co-localized with GFAP^+^ astrocytes and MAP2^+^ neurons, particularly in astrocytes, but not with CD31^+^ endothelial cells, or IBA1^+^ microglia (Supplementary Fig. 1A-H). Further, MC4R expression on astrocytes was progressively reduced during the course of EAE (Supplementary Fig. 1I). During the peak period of EAE, immunofluorescence staining confirmed a significant reduction of astrocytic MC4R in the EAE group compared with CFA group, while EA at ST36 markedly attenuated the reduction (Fig. 1K-M). Collectively, these findings indicated that EA at ST36 upregulated the expression of α-MSH and astrocytic MC4R in the spinal cord of EAE, suggesting that the therapeutic effects of EA might involve in the α-MSH-MC4R system.

### MC4R mediated the protective effect of EA at ST36 on the BBB in EAE

**Fig. 2:**
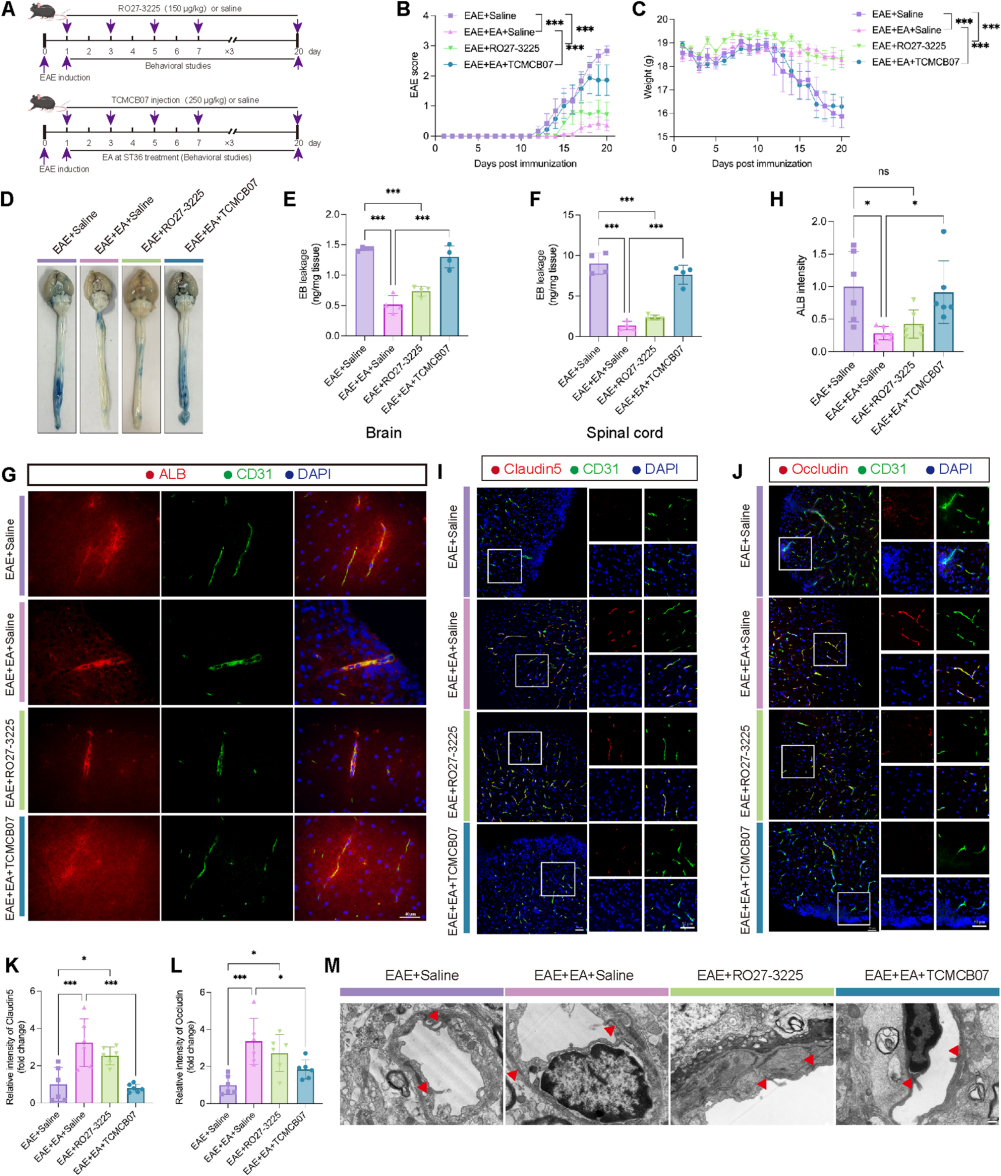
MC4R mediated the protective effect of EA at ST36 on the BBB in EAE. (**A**) Schematic diagram showing the treatment of RO27-3225 and TCMCB07 in mice. (**B**) The EAE score of four groups. (**C**) The body weight of four groups. *n* = 5. (**D**) Representative images of the leakage of EB. (**E**) The statistical results of EB leakage into the brain. (**F**) The statistical results of EB leakage into the spinal cord. *n* = 4. (**G**) Representative images of the leakage of ALB in the spinal cord. Scale bar: 40 μm. (**H**) The statistical results of ALB intensity in G. *n* = 3. (**I**) Representative images of Claudin5 and CD31 of immunofluorescence staining. Scale bar: 50 μm. (**J**) Representative images of Occludin and CD31 of immunofluorescence staining. Scale bar: 50 μm. (**K**) The statistical results of Claudin5 intensity in I. (**L**) The statistical results of Occludin intensity in J. *n* = 3. (**M**) Representative TEM images showing the ultrastructure of TJs. Red arrowheads, TJs. Scale bar: 400 nm. Two-way ANOVA is used in B, C. One-way ANOVA is used in E, F, H, K, L

To investigate whether MC4R is involved in the therapeutic effects of EA, we administered the MC4R agonist RO27-3225 and the antagonist TCMCB07 (Fig. 2A). Compared with the EAE + Saline group, both the EAE + EA + Saline and EAE + RO27-3225 groups exhibited significantly reduced clinical score and delayed disease onset, whereas the EAE + EA + TCMCB07 group showed significantly elevated clinical score relative to the EAE + EA + Saline group (Fig. 2B). Consistently, weight loss was pronounced in the EAE + Saline and EAE + EA + TCMCB07 groups but was less severe in the EAE + EA + Saline and EAE + RO27-3225 groups (Fig. 2C). These results indicated that MC4R activation significantly alleviated disease severity, while TCMCB07 diminished the protective effect of EA.

We next evaluated BBB integrity on day 15 post-immunization. EB leakage was significantly reduced in the EAE + EA + Saline and EAE + RO27-3225 groups compared with EAE + Saline group, but markedly increased in the EAE + EA + TCMCB07 group relative to the EAE + EA + Saline group (Fig. 2D-F). Immunofluorescence staining for ALB yielded consistent results (Fig. 2G-H). Furthermore, tight junctions (TJs) Claudin5 and Occludin were significantly upregulated on endothelial cells in EAE + EA + Saline and EAE + RO27-3225 groups, but were markedly reduced in EAE + Saline and EAE + EA + TCMCB07 groups (Fig. 2I-L). Further TEM analysis confirmed these findings: continuous and intact TJs with high electron density were observed in EAE + EA + Saline and EAE + RO27-3225 groups, whereas sparse, disrupted, or absent TJs were evident in EAE + Saline and EAE + EA + TCMCB07 groups (Fig. 2M). Taken together, these results indicated that EA at ST36 and pharmacological activation of MC4R preserved BBB integrity in EAE, while pharmacological blockade of MC4R abrogated this protective effect of EA.

### MC4R agonist alleviated neuroinflammation and demyelination, whereas MC4R antagonist attenuated the therapeutic effect of EA

**Fig. 3:**
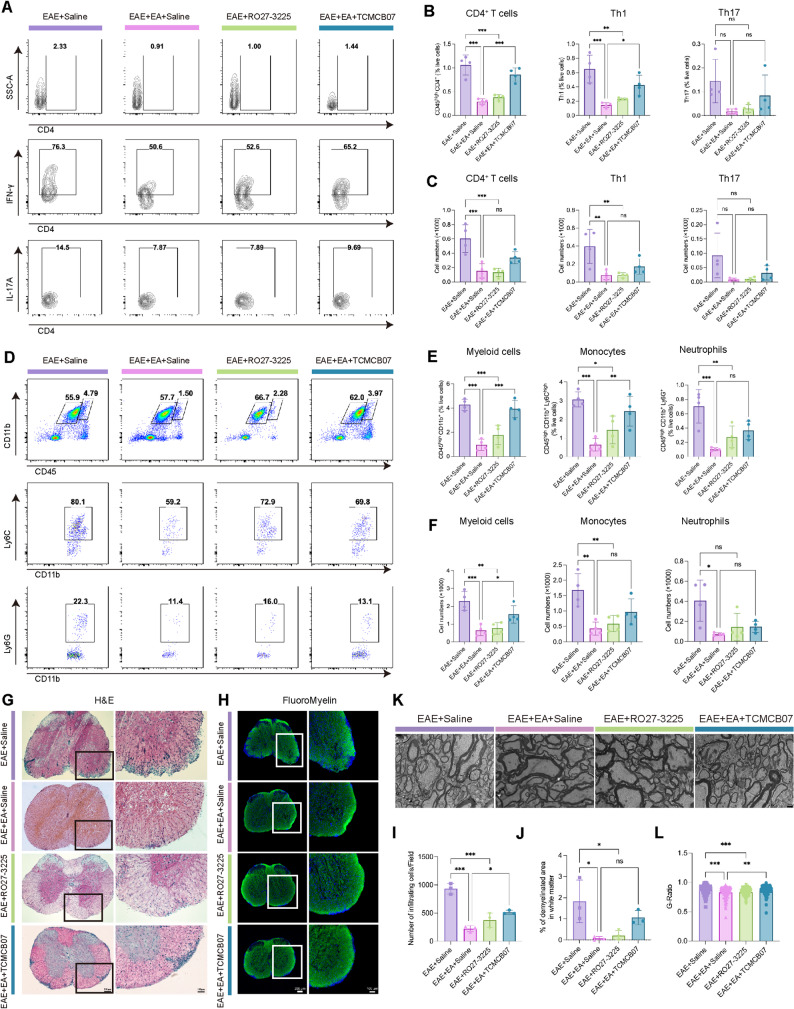
MC4R agonist alleviated the neuroinflammation and demyelination, while MC4R antagonist weakened the therapeutic effect of EA. (**A**) Representative images of flow cytometric analysis of CD4^+^ T cells (CD45^high^ CD4^+^), Th1 (CD45^high^ CD4^+^ IFNγ^+^) and Th17 (CD45^high^ CD4^+^ IL17A^+^). (**B-C**) Statistical analysis of the percentage and absolute count of the data in A. *n* = 4. (**D**) Representative images of flow cytometric analysis of myeloid cells (CD45^high^ CD11b^+^), monocytes (CD45^high^ CD11b^+^ Ly6C^high^) and neutrophils (CD45^high^ CD11b^+^ Ly6G^+^) in the CNS of mice. (**E-F**) Statistical analysis of the percentage and absolute count of the data in D. *n* = 4. (**G**) Representative images of H&E staining. (**H**) Representative images of FluoroMyelin staining. Scale bars: 200 μm (left panel), 100 μm (right panel). (**I**) Quantification of the number of infiltrating cells in G. (**J**) Quantification of the demyelinated area in white matter in H. *n* = 4. (**K**) Representative TEM images showing the ultrastructure of myelin sheaths. Scale bar: 1 μm. (**L**) G-ratio of myelinated fiber in K (*n* ≥ 100). One-way ANOVA is used in B, C, E, F, I, J, L

We assessed the infiltration of lymphocytes and myeloid cells in CNS by FCM on day 15 post-immunization, with the gating strategy shown in Supplement Fig. 2. Compared with the EAE + Saline group, the proportion and absolute number of lymphocytes and myeloid cells of EAE + EA + Saline and EAE + RO27-3225 groups decreased significantly. However, the proportion of CD4^+^ T cells and Th1 cells, as well as the proportion and absolute number of myeloid cells and monocytes in EAE + EA + TCMCB07 group significantly increased compared with EAE + EA + Saline group, with no significant changes in the absolute number of CD4^+^ T cells and Th1 cells. The proportion and absolute number of Th17 and neutrophils did not differ significantly between EAE + EA + TCMCB07 and EAE + EA + Saline groups (Fig. 3A-F).

Histological examination revealed parallel changes in spinal cord pathology. H&E staining and FluoroMyelin™ green staining were peformed to examine the inflammatory cell infiltration and demyelination, respectively. Compared with EAE + Saline group, inflammatory cell infiltration and demyelination were significantly reduced in EAE + EA + Saline and EAE + RO27-3225 groups, whereas they were significantly exacerbated in EAE + EA + TCMCB07 group relative to EAE + EA + Saline group (Fig. 3G-J). TEM further confirmed these findings: the myelin sheath in EAE + EA + Saline and EAE + RO27-3225 groups was obviously thicker compared with EAE + Saline group, whereas it was obviously thinner in EAE + EA + TCMCB07 group compared with EAE + EA + Saline group (Fig. 3K-L).

To assess inflammatory signal at the molecular level in the spinal cord, qPCR was performed on day 15 post-immunization. The expression of most inflammatory mediators was significantly downregulated in the EAE + EA + Saline and EAE + RO27-3225 groups compared with EAE + Saline group, but upregulated in the EAE + EA + TCMCB07 group compared with EAE + EA + Saline group (Supplement Fig. 3A-N). Together, these results demonstrated that EA at ST36 and pharmacological activation of MC4R effectively alleviated the neuroinflammation and demyelination in EAE, whereas MC4R inhibition counteracted the therapeutic effect of EA.

### Astrocytic MC4R Silencing attenuated the protective effect of EA on BBB integrity in EAE

**Fig. 4:**
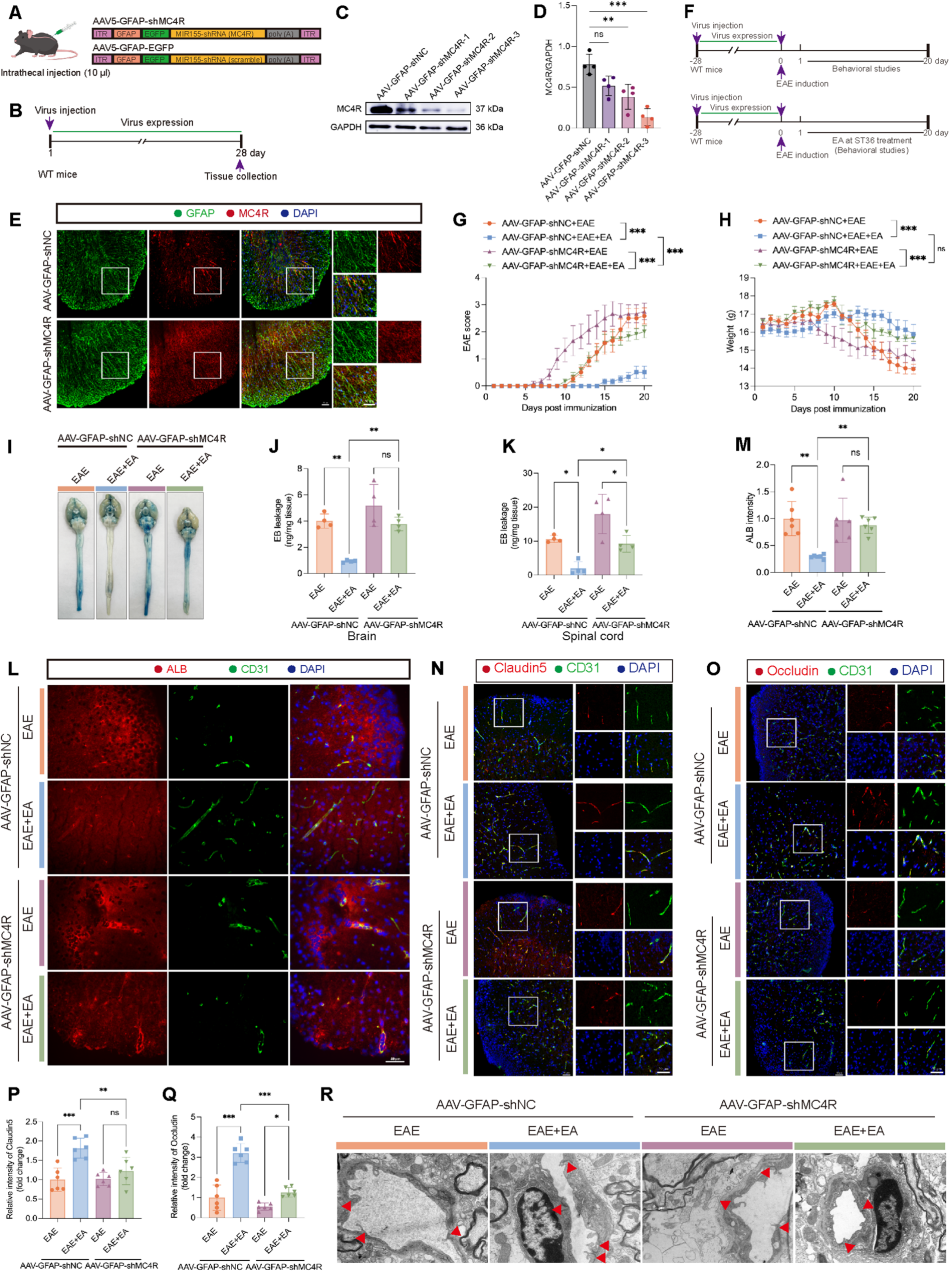
Astrocytic MC4R silencing attenuated the protective effect of EA on BBB integrity in EAE. (**A**) Schematic diagram showing the AAV construction (**B**) Schematic diagram showing the treatment of AAV in WT mice. (**C**) WB analysis of the expression of MC4R after AAV injection. (**D**) The statistical results of MC4R expression in C. *n* = 4. (**E**) Representative images of MC4R and GFAP of immunofluorescence staining. Scale bar: 50 μm. (**F**) Schematic diagram showing the treatment of AAV and the induction of EAE. (**G**) The EAE score of four groups. (**H**) The body weight of four groups. *n* = 5. (**I**) Representative images of the leakage of EB. (**J**) The statistical results of EB leakage into the brain. (**K**) The statistical results of EB leakage into the spinal cord. *n* = 4. (**L**) Representative images of the leakage of ALB in the spinal cord. Scale bar: 40 μm. (**M**) The statistical results of ALB intensity in L. *n* = 3. (**N**) Representative images of Claudin5 and CD31 of immunofluorescence staining. (**O**) Representative images of Occludin and CD31 of immunofluorescence staining. Scale bar: 50 μm. (**P**) The statistical results of Claudin5 intensity in N. (**Q**) The statistical results of Occludin intensity in O. *n* = 3. (**R**) Representative TEM images showeing the ultrastructure of TJs. Red arrowheads, TJs. Scale bar: 400 nm. Two-way ANOVA is used in G, H. One-way ANOVA is used in D, J, K, M, P, Q

To further elucidate the role of astrocytic MC4R in the therapeutic effect of EA, we selectively silenced MC4R in astrocytes using AAV vector (Fig. 4A). Spinal cord tissue was collected 28 days after intrathecal injection of AAV (Fig. 4B). The WB results showed that both AAV-GFAP-shMC4R-2 and AAV-GFAP-shMC4R-3 significantly downregulated MC4R protein levels, with AAV-GFAP-shMC4R-3 exhibiting higher knockdown efficiency and thus chosen for subsequent experiments (Fig. 4C-D). Immunofluorescence staining confirmed that the AAV specifically targeted astrocytes (Supplement Fig. 4A), and that AAV-GFAP-shMC4R-3 effectively decreased astrocytic MC4R expression (Fig. 4E). Importantly, EB and ALB leakage revealed that no BBB disruption in WT mice injected with in AAV-GFAP-shNC or AAV-GFAP-shMC4R, indicating that AAV vector itself did not impair BBB integrity (Supplementary Fig. 4B-E).

Following 28 days of AAV administration, EAE was induced (Fig. 4F). Clinical assessments showed that MC4R silencing abolished the therapeutic effect of EA: EA no longer significantly reduced the clinical score or prevented weight loss in the AAV-GFAP-shMC4R + EAE + EA group. In addition, MC4R silencing accelerated the onset of EAE to day 6 post-immunization, along with accelerated disease progression and significant weight loss (Fig. 4G-H). BBB permeability was assessed on day 15 post-immunization. In the AAV-GFAP-shNC + EAE + EA group, EA significantly reduced the leakage of EB in the brain and spinal cord. However, after MC4R silencing, EA failed to suppress EB leakage in the brain and only partially reduced leakage in the spinal cord, with significantly higher EB levels compared with AAV-GFAP-shNC + EAE + EA group (Fig. 4I-K). Immunofluorescence staining for ALB yielded similar results, showing markedly increased leakage in the AAV-GFAP-shMC4R + EAE + EA group compared with the AAV-GFAP-shNC + EAE + EA group (Fig. 4L-M).

Analysis of TJs further supported these findings. In the AAV-GFAP-shNC + EAE + EA group, EA treatment enhanced the expression of Claudin5 and Occludin, whereas this effect was abolished by MC4R silencing, with fluorescence intensity markedly reduced in the AAV-GFAP-shMC4R + EAE + EA group (Fig. 4N-Q). Consistently, TEM showed that TJs were continuous, complete and clear band structure in AAV-GFAP-shNC + EAE + EA group, while TJs were sparse or unclear in the other three groups (Fig. 4R). Together, these results indicated that astrocytic MC4R was indispensable for the protective effect of EA on BBB integrity during EAE.

### Astrocytic MC4R Silencing abolished the regulatory effects of EA on neuroinflammation and demyelination in EAE

**Fig. 5:**
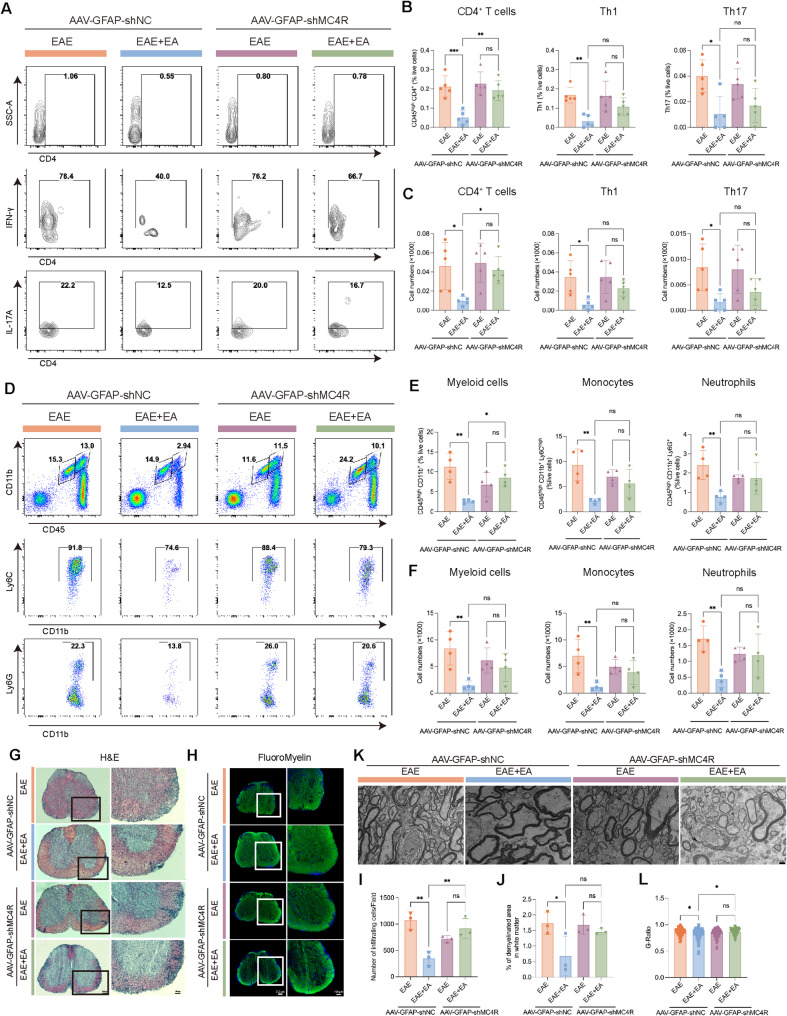
Astrocytic MC4R silencing abolished the regulatory effects of EA on neuroinflammation and demyelination in EAE. (**A**) Representative images of flow cytometric analysis of CD4^+^ T cells (CD45^high^ CD4^+^), Th1 (CD45^high^ CD4^+^ IFNγ^+^) and Th17 (CD45^high^ CD4^+^ IL17A^+^). (**B-C**) Statistical analysis of the percentage and absolute count of the data in A. *n* = 4. (**D**) Representative images of flow cytometric analysis of myeloid cells (CD45^high^ CD11b^+^), monocytes (CD45^high^ CD11b^+^ Ly6C^high^) and neutrophils (CD45^high^ CD11b^+^ Ly6G^+^). (**E-F**) Statistical analysis of the percentage and absolute count of the data in D. *n* = 4. (**G**) Representative images of H&E staining. (**H**) Representative images of FluoroMyelin staining. Scale bars: 200 μm (left panel), 100 μm (right panel). (**I**) Quantification of the number of infiltrating cells in G. (**J**) Quantification of the demyelinated area in white matter in H. *n* = 3. (**K**) Representative TEM images showing the ultrastructure of myelin sheaths. Scale bar: 1 μm. (**L**) G-ratios of myelinated fiber in K (*n* ≥ 100). One-way ANOVA is used in B, C, E, F, I, J, L

To evaluate whether astrocytic MC4R contributed to the anti-inflammatory effects of EA, we examined CNS immune cell infiltration by FCM on day 15 post-immunization. Following MC4R silencing, EA treatment failed to significantly affect lymphocytes and myeloid cells. Further comparison revealed that, compared with AAV-GFAP-shNC + EAE + EA group, the proportion and absolute number of CD4^+^ T cells, as well as the proportion of myeloid cells increased significantly in AAV-GFAP-shMC4R + EAE + EA group (Fig. 5A-F).

Histological analysis corroborated these findings. EA failed to alleviate inflammatory cell infiltration and demyelination after silencing MC4R. Conversely, the AAV-GFAP-shMC4R + EAE + EA group exhibited more severe pathology compared with AAV-GFAP-shNC + EAE + EA group (Fig. 5G-J). TEM further confirmed that the myelin sheath thickness preserved normal only in AAV-GFAP-shNC + EAE + EA group (Fig. 5K-L). At the molecular level, qPCR analysis demonstrated that the downregulation of inflammatory mediators normally induced by EA was markedly weakened after MC4R silencing. Moreover, inflammatory gene expression levels were significantly higher in AAV-GFAP-shMC4R + EAE + EA group than in the AAV-GFAP-shNC + EAE + EA group (Supplement Fig. 5A-N). Collectively, these results indicated that astrocytic MC4R was essential for the anti-inflammatory and prevening demyelination of EA in EAE.

### EA at ST36 inhibited MAPK and NF-κB signaling pathways via MC4R activation during the treatment of EAE

**Fig. 6:**
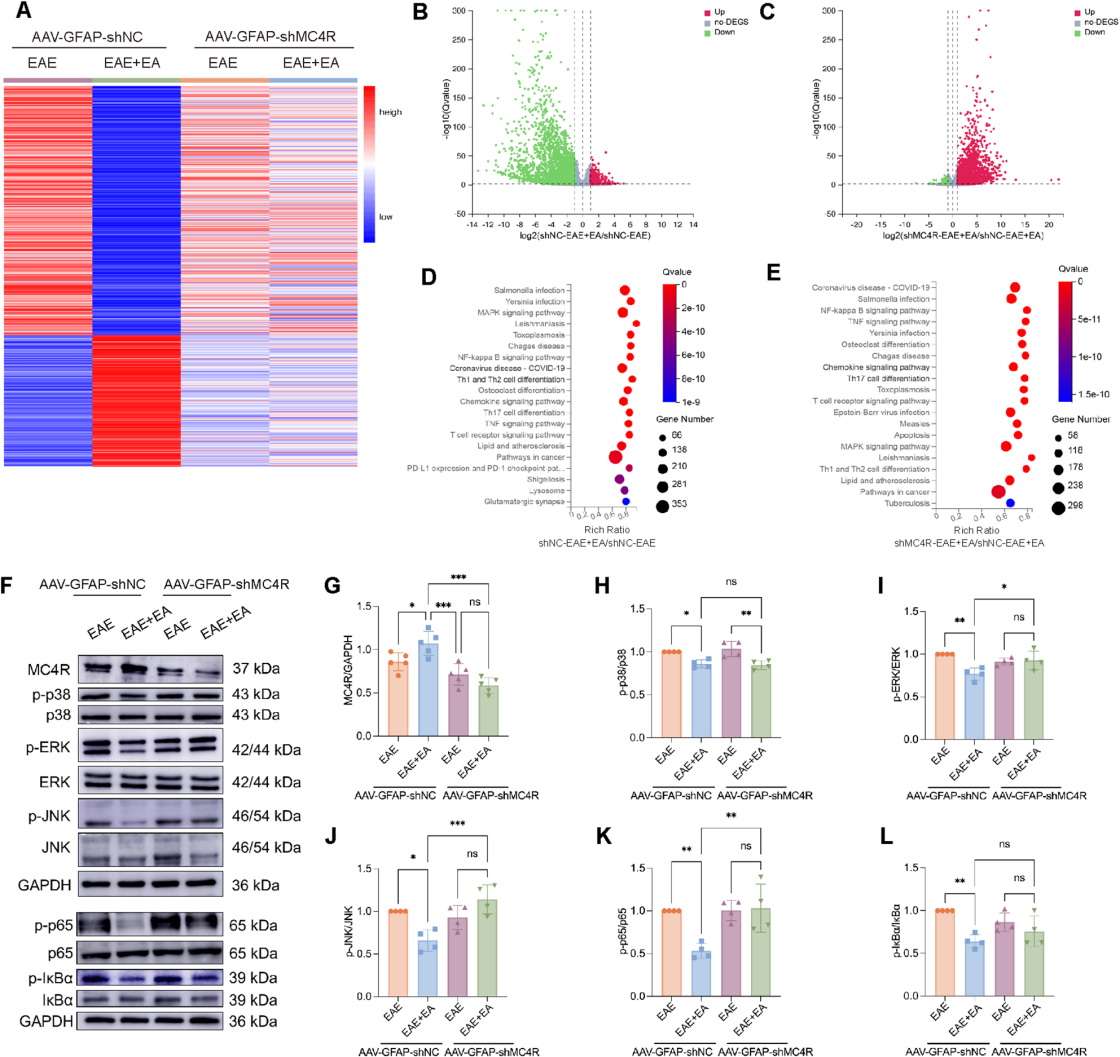
EA at ST36 inhibited MAPK and NF-κB signaling pathways via MC4R activation during the treatment of EAE. (**A**) The heatmap of DEGs with FDR ≤ 0.001 or Q value ≤ 0.05 from RNA-seq analysis of AAV-GFAP-shNC and AAV-GFAP-shMC4R groups. (**B**) Volcano plot showing the results of DEGs analysis between AAV-GFAP-shNC-EAE + EA and AAV-GFAP-shNC-EAE groups. (**C**) Volcano plot showing the results of DEGs analysis between AAV-GFAP-shMC4R-EAE + EA and AAV-GFAP-shNC-EAE + EA groups. Red represents up-regulated DEGs, blue represents down-regulated DEGs, and gray represents non-DEGs. Q value ≤ 0.05, log2 ≥ 1. (**D**) The most enriched differentially regulated KEGG pathways between AAV-GFAP-shNC-EAE + EA and AAV-GFAP-shNC-EAE groups. (**E**) The most enriched differentially regulated KEGG pathways between AAV-GFAP-shMC4R-EAE + EA and AAV-GFAP-shNC-EAE + EA groups. The bubble size indicates the number of differential genes annotated on a KEGG term, the color represents the enrichment significance value, and the redder the color, the smaller the significance value. *n* = 4/group. (**F**) WB analysis of the expression of MC4R, MAPK and NF-κB signal pathways. (**G-L**) The statistical results of MC4R, MAPK and NF-κB signal pathways proteins in F. *n* = 4. One-way ANOVA is used in G-L

To elucidate the molecular regulatory mechanism involving MC4R in EA- mediated treatment of EAE, we performed RNA-seq analysis of spinal cord tissue collected during the peak period of EAE. Differentially expressed genes (DEGs) were identified between AAV-GFAP-shNC-EAE + EA and AAV-GFAP-shNC-EAE groups, revealing 4,956 upregulated and 5,453 downregulated genes (Q value ≤ 0.05, log2 ≥ 1) (Fig. 6A-B). Similarly, comparison of AAV-GFAP-shMC4R-EAE + EA and AAV-GFAP-shNC-EAE + EA groups identified 4383 upregulated and 3601 downregulated genes (Q value ≤ 0.05, log2 ≥ 1) (Fig. 6A, C). KEGG enrichment analysis showed that in both comparisons, the most significant enriched pathways were consistently associated with MAPK and NF-κB signaling pathways, infection, immune response and lysosomes (Fig. 6D-E). These findings suggested that MC4R played a key role in regulating astrocyte-driven inflammation and mediating the therapeutic effects of EA in EAE. GO enrichment analysis further showed that the most significant enriched biological processes were related to inflammatory signal factor production, signal transduction, cell migration and apoptosis (Supplementary Fig. 6A-B). Collectively, these data indicated that MC4R silencing aggravated CNS inflammatory in EA-treated mice, supporting the view that EA exerted CNS protection by activating astrocytic MC4R in spinal cord.

To verify the RNA-seq results, we assessed MAPK and NF-κB signaling pathways via WB analysis. Compared with AAV-GFAP-shNC + EAE group, MC4R expression was significantly upregulated in the AAV-GFAP-shNC + EAE + EA group, but markedly reduced following MC4R silencing (Fig. 6F-G). In the AAV-GFAP-shNC + EAE + EA group, EA significantly inhibited MAPK and NF-κB pathway activity, as reflected by decreased phosphorylation of p38, ERK, JNK, p65 and IκBα. However, after MC4R silencing, EA treatment failed to significantly reduce the levels of these proteins, except for p-p38. Notably, the protein levels of p-ERK, p-JNK, and p-p65 were significantly upregulated in the AAV-GFAP-shMC4R + EAE + EA group compared with the AAV-GFAP-shNC + EAE + EA group (Fig. 6F, H-L). Together, these results indicated that EA at ST36 inhibited MAPK and NF-κB signaling pathways by activating MC4R during the treatment of EAE.

### Role and mechanism of MC4R in inflammatory-injured astrocytes

We established an in vitro model of LPS-induced astrocyte damage. qPCR results showed that LPS treatment for 1 h significantly upregulated inflammatory factors, reaching peak levels after 4 h of exposure. While α-MSH effectively attenuated inflammatory injury in astrocytes after 1-h LPS stimulation, neither α-MSH nor RO27-3225 could significantly mitigate such injury following 4-h LPS exposure (Supplementary Fig. 7A-C).

**Fig. 7:**
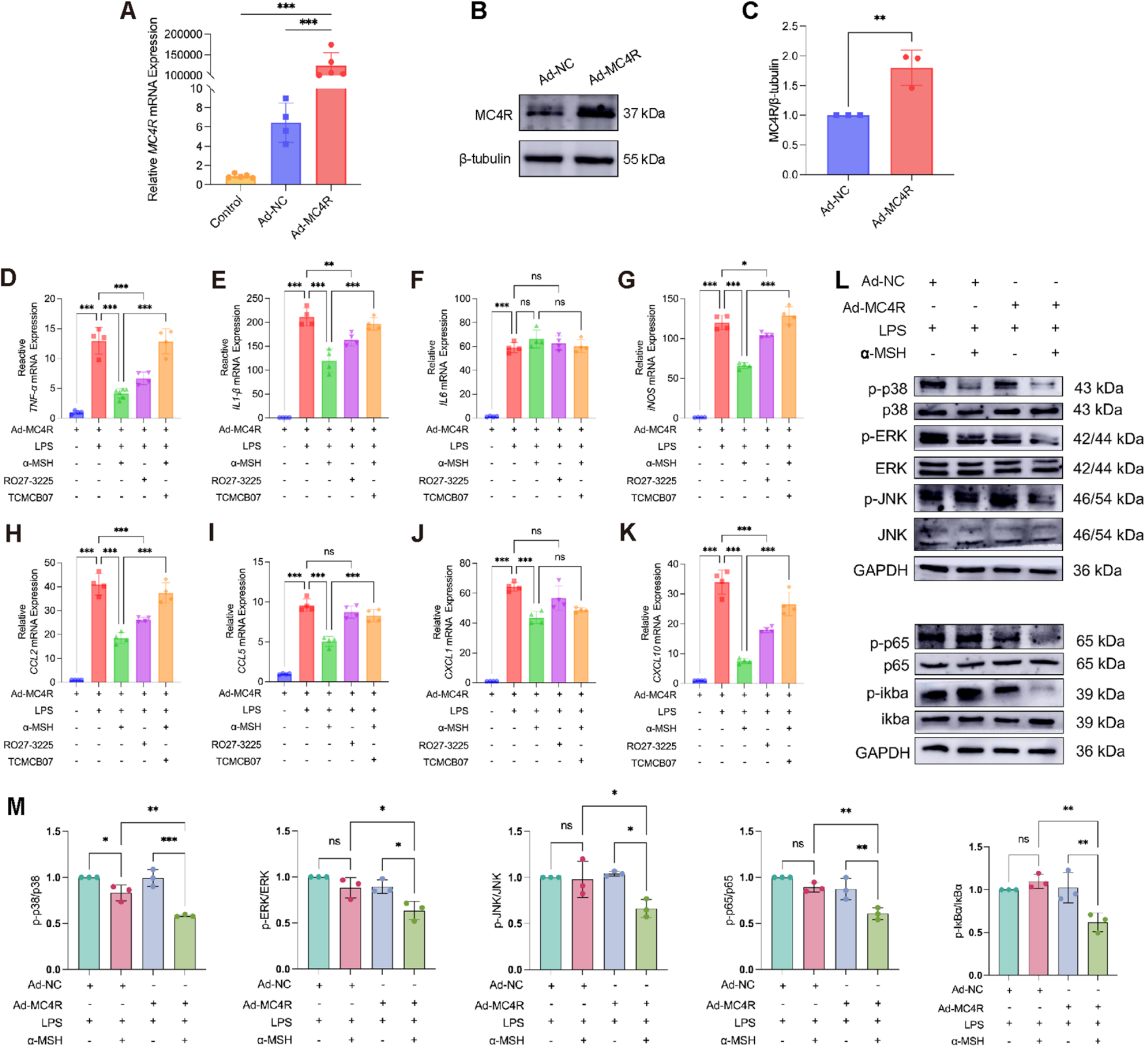
Role and mechanism of MC4R in inflammatory injured astrocytes. (**A**) qPCR analysis of the mRNA expression of *MC4R*. *n* ≥ 4. (**B**) WB analysis of the expression of MC4R. (**C**) The statistical results of MC4R in B. *n* = 3. (**D-K**) qPCR analysis of the mRNA expression of *TNF-α*, *IL-1β*, *IL-6*, *iNOS*, *CCL2*, *CCL5*, *CXCL1*, *CXCL10*. *n* = 4. (**L**) WB analysis of the expression of MAPK and NF-κB signal pathways. (**M**) The statistical results of MAPK and NF-κB signal pathway proteins in L. *n* = 3. One-way ANOVA is used in A, D-K, M. *t*-test is used in C

Given that EA at ST36 significantly upregulated MC4R expression in the spinal cord, we hypothesized that overexpression of MC4R in primary astrocytes might augment the reactivity to α-MSH, thus alleviate the inflammatory damage. Therefore, we transduced astrocytes with an adenoviral vector to overexpress MC4R. It was confirmed that the best transfection condition was transfection with EIS (MOI = 10) for 72 h (Supplementary Fig. 8A). Then, qPCR and WB verified successful MC4R overexpression in the Ad-MC4R group compared with the Ad-NC group (Fig. 7A-C). qPCR results showed that α-MSH significantly downregulated the expression of *TNF-α*, *IL-1β*, *iNOS*, *CCL2*, *CCL5*, *CXCL1* and *CXCL10*, which was markedly attenuated after the application of TCMCB07. Similarly, RO27-3225 also significantly downregulated the expression of *TNF-α*, *IL-1β*, *iNOS*, *CCL2* and *CXCL10* (Fig. 7D-K). These results indicated that MC4R agonists inhibited inflammation in MC4R-overexpressing astrocytes after the stimulation of LPS for 4 h.

**Fig. 8:**
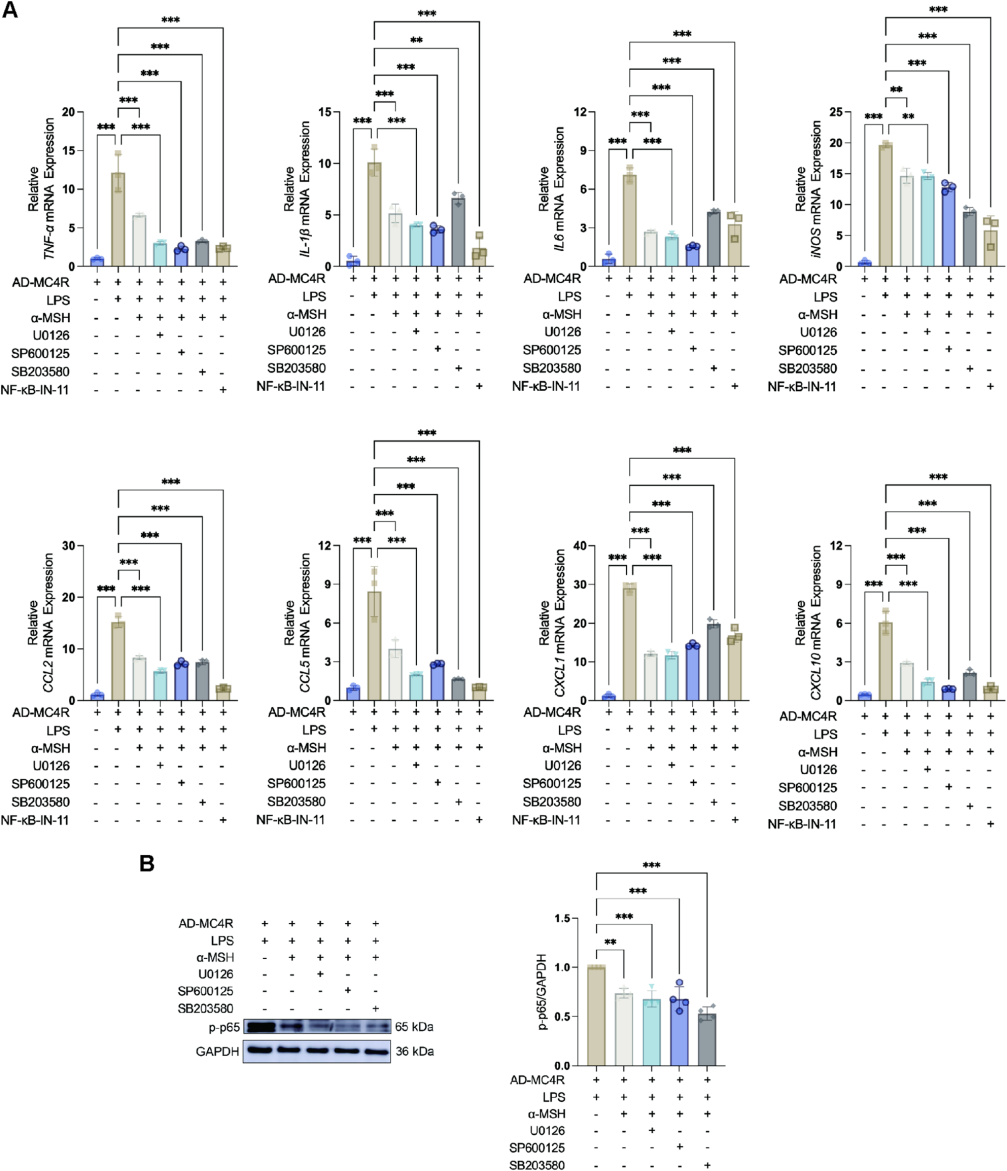
Effect of α-MSH on LPS-induced expression of pro-inflammatory mediators in the presence of U0126, SP600125, SB203580, or NF-kB-IN-11 and the effect of MAPK on p-p65 in astrocytes. (**A**) qPCR analysis of the mRNA expression of *TNF-α*, *IL-1β*, *IL-6*, *iNOS*, *CCL2*, *CCL5*, *CXCL1*, *CXCL10*. *n* = 3. (**B**) WB analysis of the expression of p-p65 normalized to GAPDH. *n* = 4. One-way ANOVA is used in A-B

To validate the underlying signaling mechanisms, MAPK and NF-κB signaling pathways were investigated. In MC4R-overexpressing astrocytes, α-MSH treatment significantly suppressed phosphorylation of p38, ERK, JNK, p65, and IκBα (Fig. 7L-M), suggesting that MC4R activation dampened both MAPK and NF-κB pathways under inflammatory conditions. Then, to determine whether inhibition of these pathways was functionally required for the anti-inflammatory effects of α-MSH-MC4R signaling, we applied pathway-specific inhibitors in LPS-stimulated astrocytes. The ERK inhibitor U0126, the JNK inhibitor SP600125, the p38 MAPK inhibitor SB203580, and the NF-kB inhibitor NF-κB-IN-11 each significantly reduced the expression of pro-inflammatory mediators in the presence of α-MSH, confirming that blockade of either MAPK or NF-κB contributed to downstream inflammatory suppression (Fig. 8A). Given prior evidence that MAPK signaling pathway was upstream of p65 [35], we then investigated whether the MAPK signaling pathway stimulated NF-kB in LPS-activated astrocytes. In LPS-stimulated astrocytes, pretreatment with each MAPK inhibitors significantly reduced p-p65 level in the presence of α-MSH (Fig. 8B). Together, these findings indicated that MC4R overexpression confered astrocytic sensitivity to α-MSH, enabling robust inhibition of MAPK/NF-κB signaling in LPS-stimulated astrocytes.

## Discussion

Neuroinflammation and BBB disruption are central events in the initiation and progression of MS. As a key component of the BBB, astrocytes actively respond to inflammatory cues by releasing chemokines and cytokines that amplify leukocyte infiltration and sustain the inflammatory cascade [36–38]. These observations underscore the importance of astrocyte-driven mechanisms in MS pathology and highlight astrocytes as promising therapeutic targets.

In the present study, we demonstrated that EA at ST36 alleviated BBB disruption and neuroinflammation in EAE and identified astrocytic MC4R as a critical mediator of these beneficial effects. Our privious studies have demonstrated that EA at ST36 suppresses of miR-155-mediated inflammatory signaling and modulates of T-cell activation and differentiation [13, 14]. Subsequently we have found that EA at ST36 promotes stem cell proliferation and oligodendroglial lineage commitment [15, 16]. Although these mechanisms initially appear heterogeneous, the core advantages of EA therapy lie in its pleiotropic and systemic effects. The anti‑inflammatory microenvironment fostered by EA may further support the survival and differentiation of endogenous neural stem cells and oligodendrocyte precursor cells, laying the groundwork for subsequent remyelination. Thus, EA therapy integrates both anti‑inflammatory and pro‑repair dual mechanisms. Our current findings extend this concept by identifying astrocytic MC4R as an additional and previously unrecognized target of EA. Rather than replacing prior mechanisms, the α-MSH-MC4R signaling likely operates in parallel with these established pathways. This distributed regulatory profile may explain why EA exerts robust efficacy in neuroinflammatory conditions such as EAE. Furthermore, future studies including a full sham EA group and exploring additional acupoints to further strengthen evidence supporting acupoint-specific actions of EA at ST36 will be an intriguing direction.

We also provided mechanistic insight into how EA regulated α-MSH and MC4R expression in the spinal cord. Prior works have shown that EA activates POMC neurons and increases the production of α-MSH [17, 18]. This neuroendocrine route offers a coherent mechanistic link between peripheral EA stimulation and enhanced α-MSH signaling within the CNS. In current study, we showed that EA significantly increased the expression of α-MSH and astrocytic MC4R in the spinal cord. Given that α‑MSH has been shown to increase MC4R expression levels [39], EA‑induced upregulation of astrocytic MC4R may be mediated through elevated α‑MSH.

We observed decreased MC4R expression in astrocytes during the acute phase of EAE, whereas Kamermans et al. [34] reported upregulated MC4R expression on astrocytes in secondary progressive MS lesions. However, alterations in MC4R during EAE pathogenesis have scarcely been documented. We postulate this may be not contradictory, rather likely reflecting differences in disease stage, lesion region, and inflammatory context. Acute EAE characterized by a highly proinflammatory milieu may downregulate MC4R expression. In chronic human MS lesions, however, prolonged inflammation may induce a compensatory upregulation of MC4R as part of an endogenous attempt to restrain glial activation.

MC4R is a G protein-coupled receptor known to activate the cAMP-PKA-CREB [40], and suppress MAPK [28, 41] and NF-κB pathways [24]. Moreover, it has been reported that MAPK signaling pathway was upstream of NF-kB [35]. These studies align with our findings that EA inhibits MAPK/NF-κB signaling via astrocytic MC4R, thereby reducing pro-inflammatory transcriptional programs. Moreover, studies also have showed that MC4R activation increases brain-derived neurotrophic factor (BDNF) expression in astrocytes [39, 40], providing a complementary neuroprotective mechanism. It has been also reported that MC4R is upregulated in spinal cord neurons following spinal cord injury, but not expressed in astrocytes. Activating MC4R promotes functional recovery by suppressing oxidative stress in spinal cord injury models predominantly characterized by neuronal damage [30], MC4R in neurons may play a primary role. Such discrepancies necessitate the development of more advanced experimental methodologies to further validate the expression dynamics and alterations of MC4R across distinct disease models.

EA orchestrates a multi-layered regulatory response across CNS and peripheral immune system. In current study, we clarified the MC4R-dependent beneficial effects of EA in CNS. The current preclinical findings provide a mechanistic foundation necessary to future clinical research. Selective MC4R agonists, including setmelanotide, may be administered to MS patients to exert immunomodulatory effects while circumventing systemic immunosuppression. Due to astrocytic heterogeneity, they may differentially respond to MC4R signaling, highlighting the need for future studies integrating multi‑omics to identify and mark the astrocyte subpopulations. Coupled with the development of novel genetic tool mice, this will enable functional dissection of MC4R roles within defined astrocyte subtypes.

## Conclusion

**Fig. 9:**
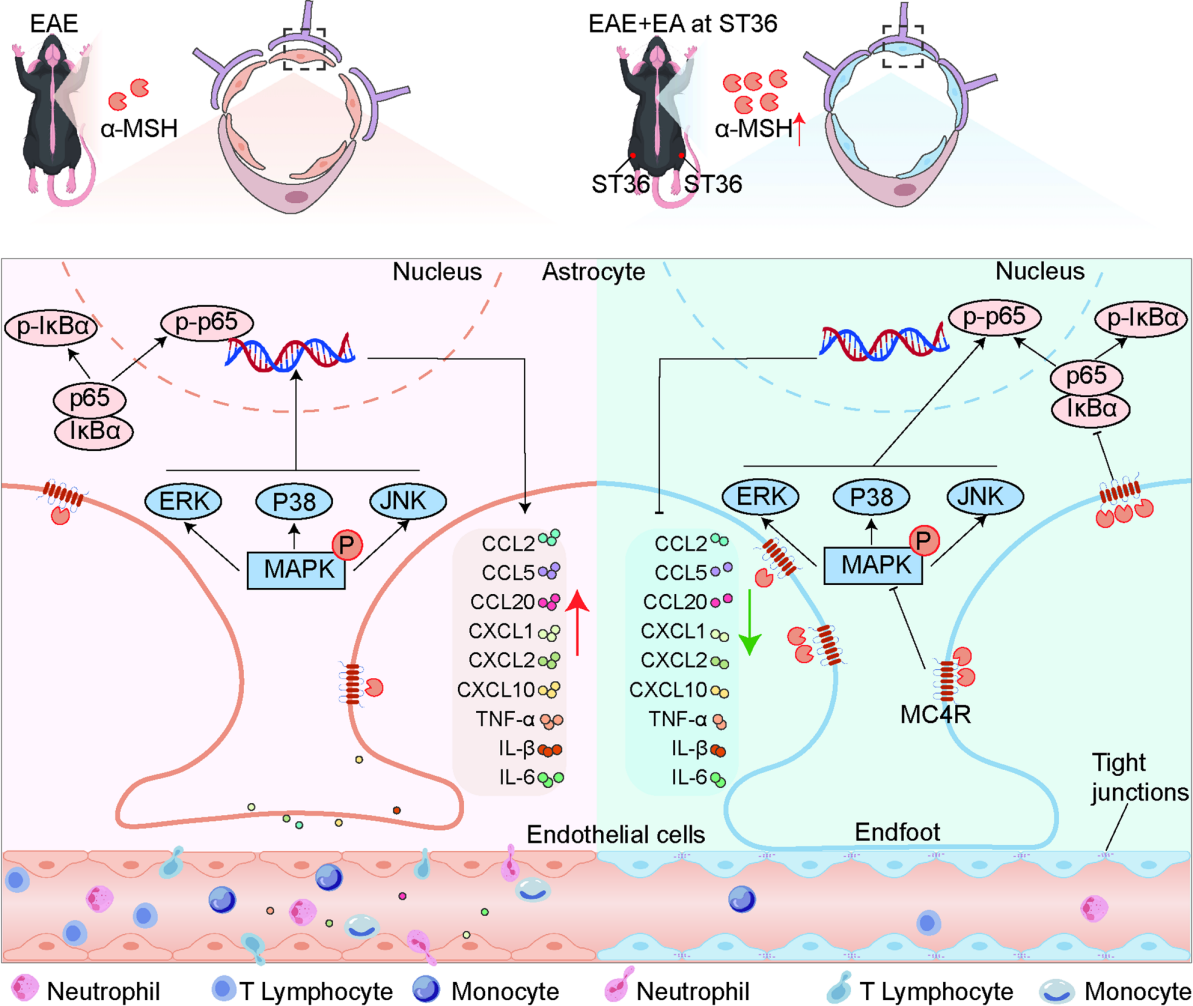
Diagram of mechanism. EA at ST36 significantly reduced BBB damage and neuroinflammation via astrocytic MC4R to inhibit MAPK and NF-κB signaling pathways in EAE mice

In summary, this study identified astrocytic MC4R signaling as a central mechanism through which EA at ST36 mitigated neuroinflammation and BBB disruption in EAE. This effect involved that EA enhanced α-MSH-MC4R activity in the spinal cord, leading to suppression of MAPK/NF-κB signaling pathways. These findings established astrocytic MC4R as a critical mediator in the neuroprotective effects of EA in EAE and suggested that targeting astrocytic MC4R could represent a promising therapeutic strategy for EAE and other neuroinflammatory diseases without systemic immunosuppression (Fig. 9).

## Supplementary Information

Supplementary Material 1.

Supplementary Material 2.

Supplementary Material 3.

Supplementary Material 4.

## Data Availability

The data supporting the results reported in the article are available on request from the corresponding author upon reasonable request.

## Abbreviations

MS: Multiple sclerosis
EA: Electroacupuncture
EAE: Experimental autoimmune encephalomyelitis
BBB: Blood-brain barrier
α-MSH: α-melanocyte-stimulating hormone
MC4R: Melanocortin-4 receptor
SPF: Special pathogen-free
AAV: Adeno-associated virus
RNA-seq: RNA-sequencing
WB: Western blot
CNS: Central nervous system
POMC: Pro-opiomelanocortin
CFA: Complete freund’s adjuvant
FBS: Fetal bovine serum
CM: Culture medium
EIS: Enhanced Infection Solution
MOI: Multiplicity of Infection
LPS: Lipopolysaccharide
qPCR: Quantitative real-time PCR
TEM: Transmission electron microscopic
EB: Evans blue
FCM: Flow cytometry
ELISA: Enzyme linked immunosorbent assay
ALB: Albumin
TJs: Tight junctions
