# Supplementary material for: Electroacupuncture alleviates blood-brain barrier disruption and neuroinflammation via astrocytic MC4R in a mouse model of multiple sclerosis
Source: J Neuroinflammation. 2025 Dec 26;23:40. doi: 10.1186/s12974-025-03667-1 (PMC12849424; doi:10.1186/s12974-025-03667-1)
Supplement: Supplementary file 3 — Supplementary Material 3. [file 12974_2025_3667_MOESM3_ESM.docx]

**Supplement file 1**

**Supplement Figures**


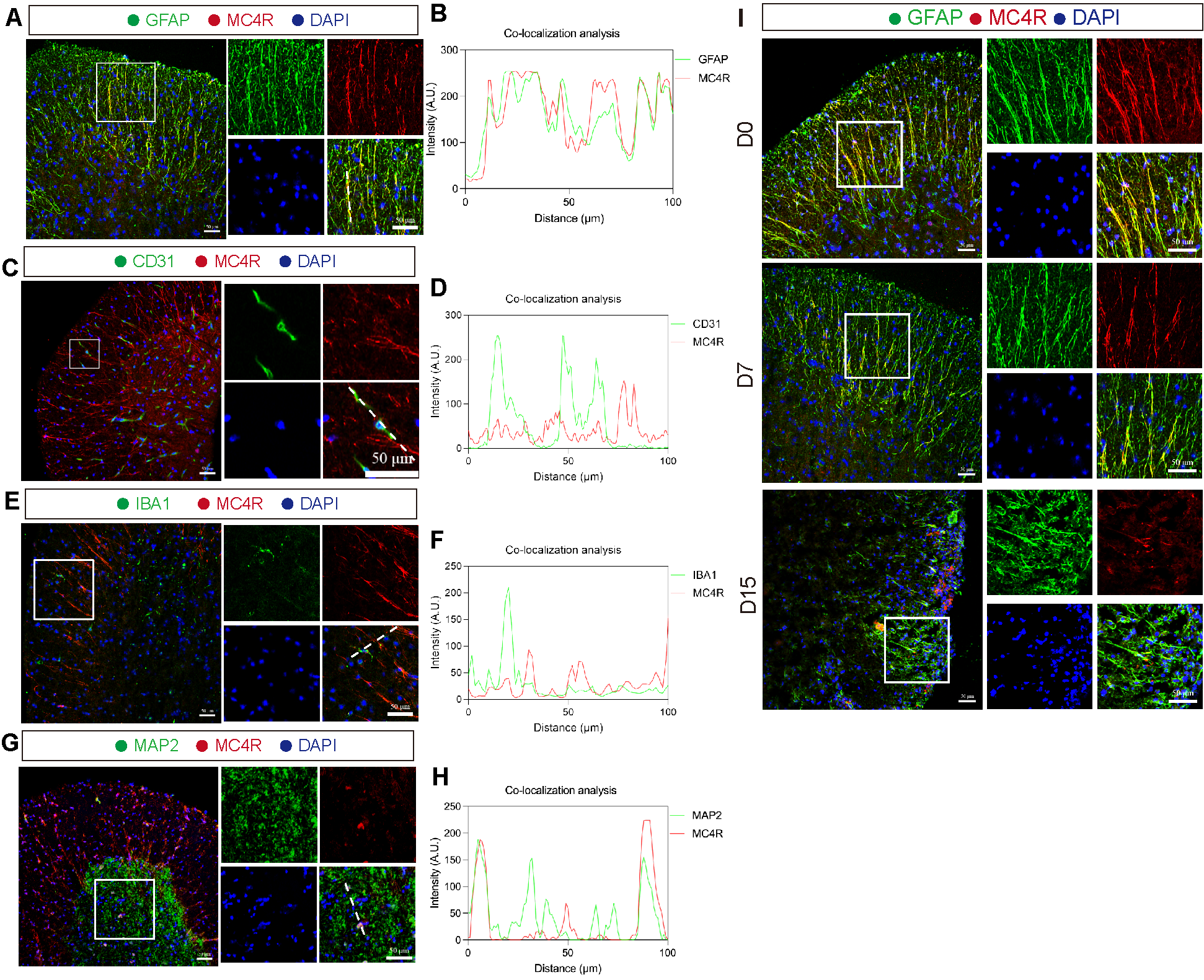


**Supplement Fig. 1.** **Localization of MC4R expression in spinal cord and the changes with EAE progress**

(A) Representative images of MC4R and GFAP by immunofluorescence staining. Scale bars: 50 μm. (B) The co-localization analysis of MC4R and GFAP. (C) Representative images of MC4R and CD31 by immunofluorescence staining. Scale bars: 50 μm. (D) The co-localization analysis of MC4R and CD31. (E) Representative images of MC4R and IBA1 by immunofluorescence staining. Scale bars: 50 μm. (F) The co-localization analysis of MC4R and IBA1. (G) Representative images of MC4R and MAP2 by immunofluorescence staining. Scale bars: 50 μm. (H) The co-localization analysis of MC4R and MAP2. (I) Representative images of MC4R and GFAP by immunofluorescence staining at different periods of EAE. Scale bars: 50 μm.


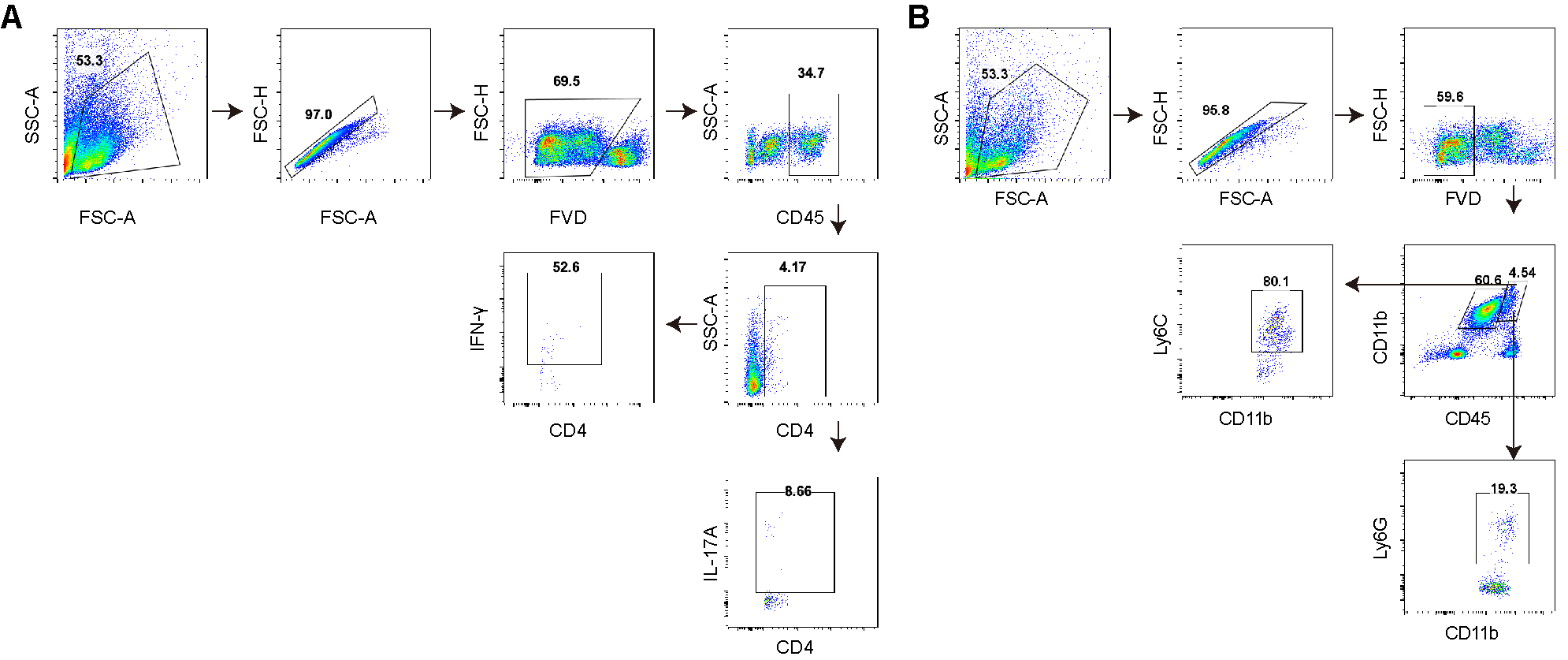


**Supplement Fig. 2. The gating strategy for central infiltration of inflammatory cells.**

The changes of CD4^+^ T lymphocytes and myeloid cells in the brain were gated according to the cell size, the complexity of the internal structure of cells, single cells and distinction between living and dead cells. (A) The gating strategy of CD4^+^ T cells (CD45^high^ CD4^+^), Th1 (CD45^high^ CD4^+^ IFNγ^+^) and Th17 (CD45^high^ CD4^+^ IL17A^+^). (B) The gating strategy of myeloid cells (CD45^high^ CD11b^+^), monocytes (CD45^high^ CD11b^+^ Ly6C^high^) and neutrophils (CD45^high^ CD11b^+^ Ly6G^+^).

**
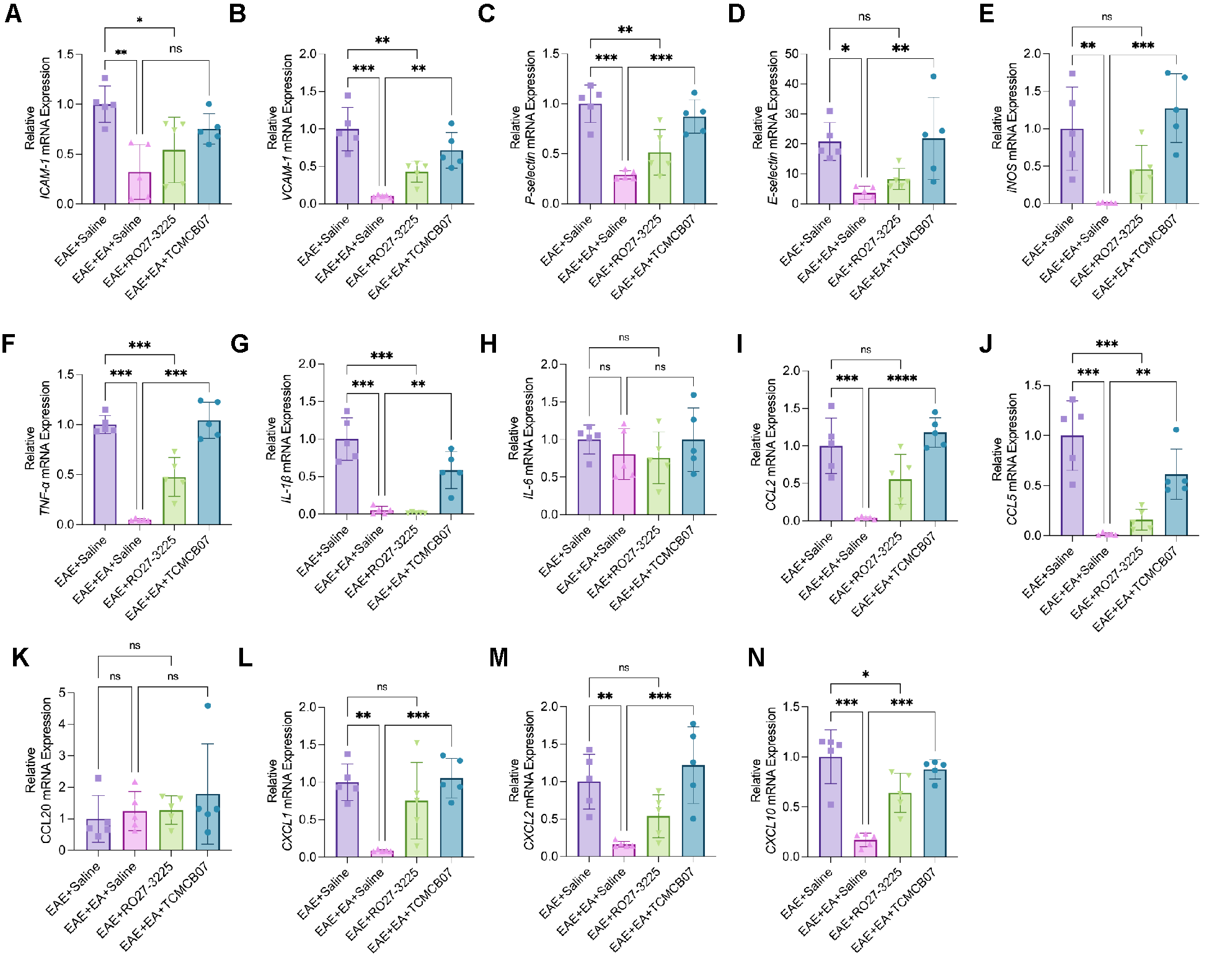
**

**Supplement Fig. 3. MC4R agonist reduced the expression of inflammatory signal molecules in spinal cord of EAE mice, while MC4R antagonist weakened the therapeutic effect of EA.**

(A-N) qPCR analysis the mRNA expression of *ICAM-1*, *VCAM-1*, *P-selectin*, *E-selectin*, *TNF-α*, *IL-1β*, *IL-6*, *iNOS*, *CCL2*, *CCL5*, *CCL20*, *CXCL1*, *CXCL2*, *CXCL10*. n = 5. One-way ANOVA is used in A-N.

**
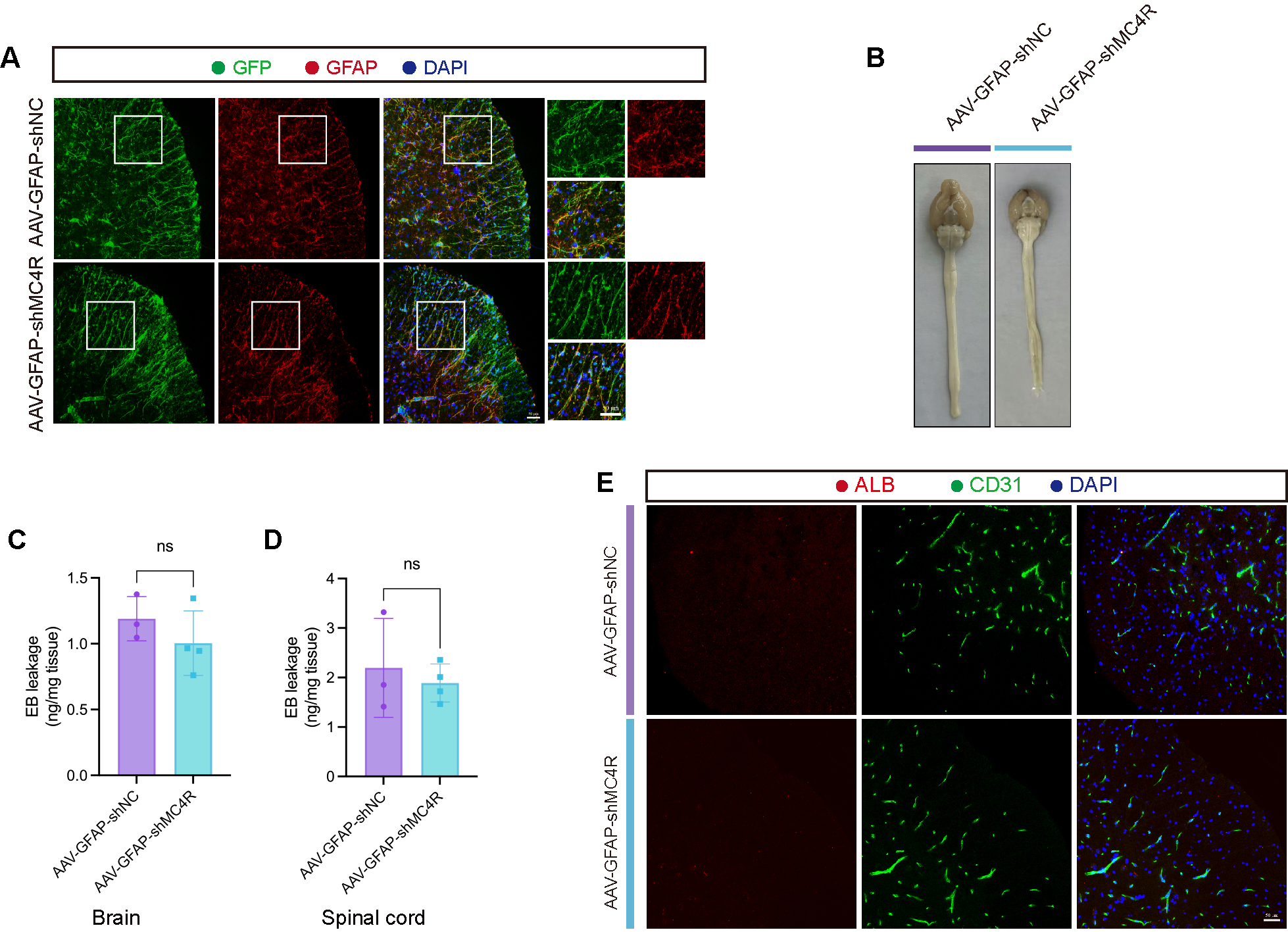
**

**Supplement Fig. 4. AAV specifically targeting astrocytes and effect of AAV on BBB permeability in WT mice.**

(A) Representative images of GFP and GFAP immunofluorescence staining. Scale bar: 50 μm. (B) Representative images showing the leakage of EB. (C) The statistical results of EB leakage into the brain. (D) The statistical results of EB leakage into the spinal cord. n ≥ 3. (E) Representative images showing the leakage of ALB in the lumbar spinal cord. Scale bar: 50 μm. *t*-test is used in B, C.


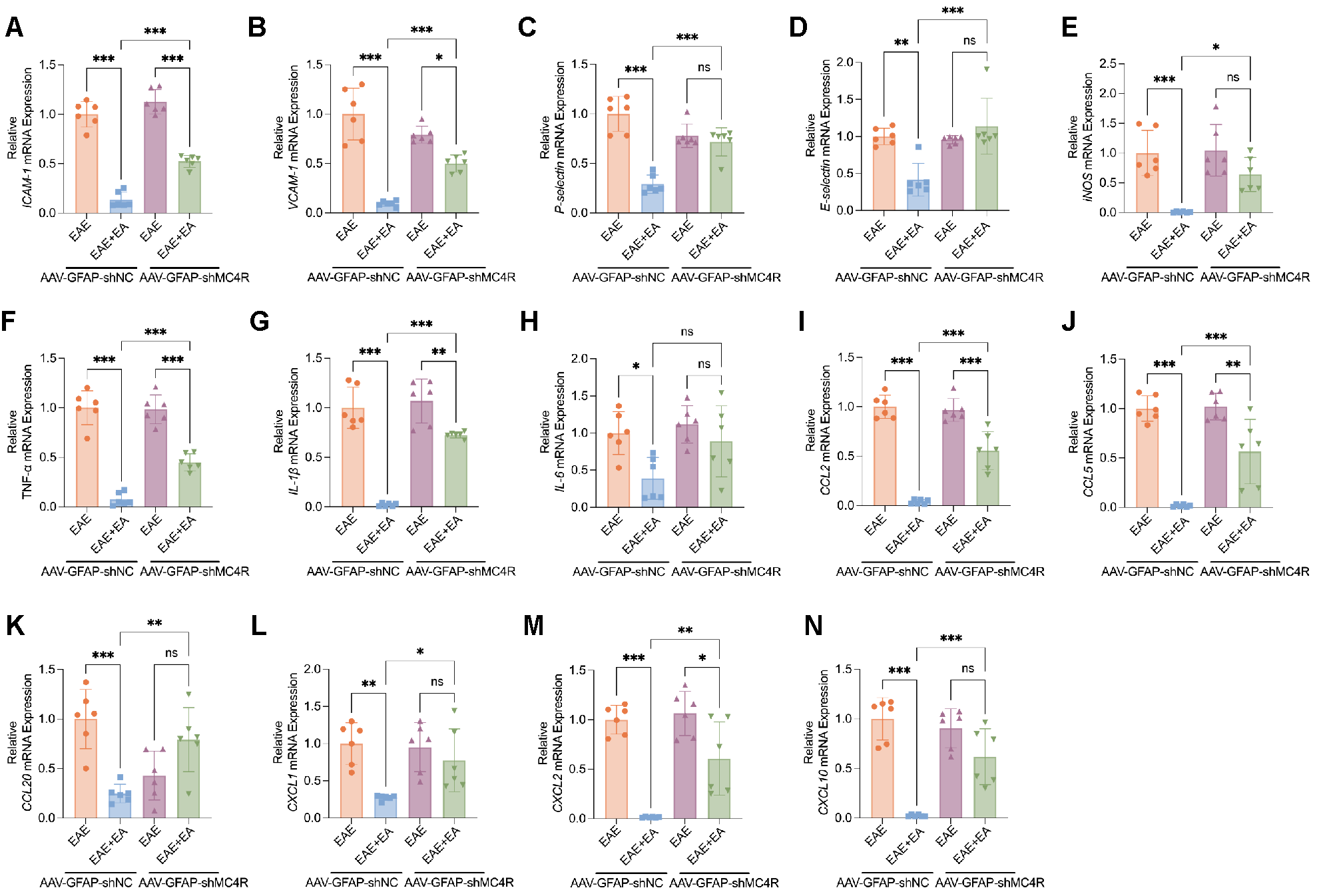


**Supplement Fig. 5. MC4R silencing weakened the regulation of EA on inflammatory signal molecules in spinal cord of EAE.**

(A-N) qPCR analysis the mRNA expression of *ICAM-1*, *VCAM-1*, *P-selectin*, *E-selectin*, *TNF-α*, *IL-1β*, *IL-6*, *iNOS*, *CCL2*, *CCL5*, *CCL20*, *CXCL1*, *CXCL2*, *CXCL10*. n = 5. One-way ANOVA is used in A-N.


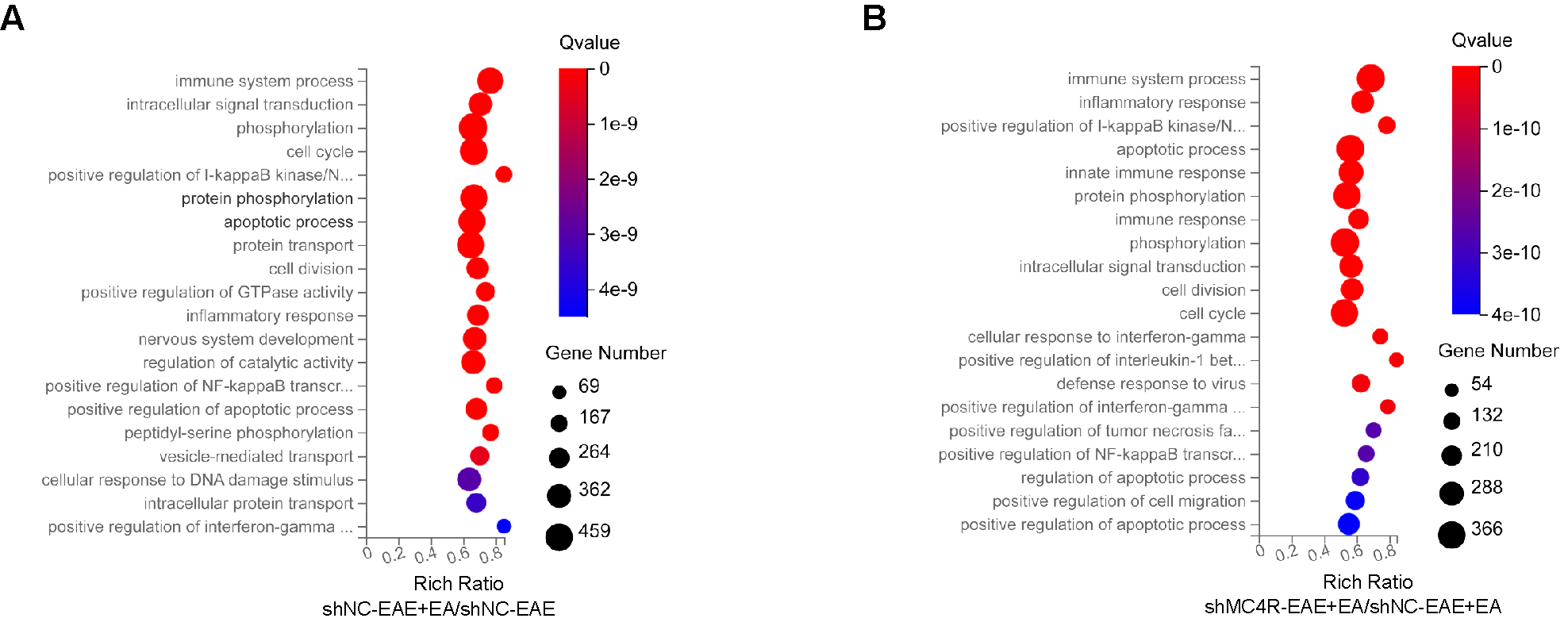


**Supplement Fig. 6. GO analysis of biological processes**

(A) The most enriched differentially regulated GO biological process between AAV-GFAP-shNC-EAE+EA and AAV-GFAP-shNC-EAE groups. (B) The most enriched differentially regulated GO biological process between AAV-GFAP-shMC4R-EAE+EA and AAV-GFAP-shNC-EAE+EA groups. The bubble size indicates the number of differential genes annotated on a GO term, the color represents the enrichment significance value, and the redder the color, the smaller the significance value. n = 4.


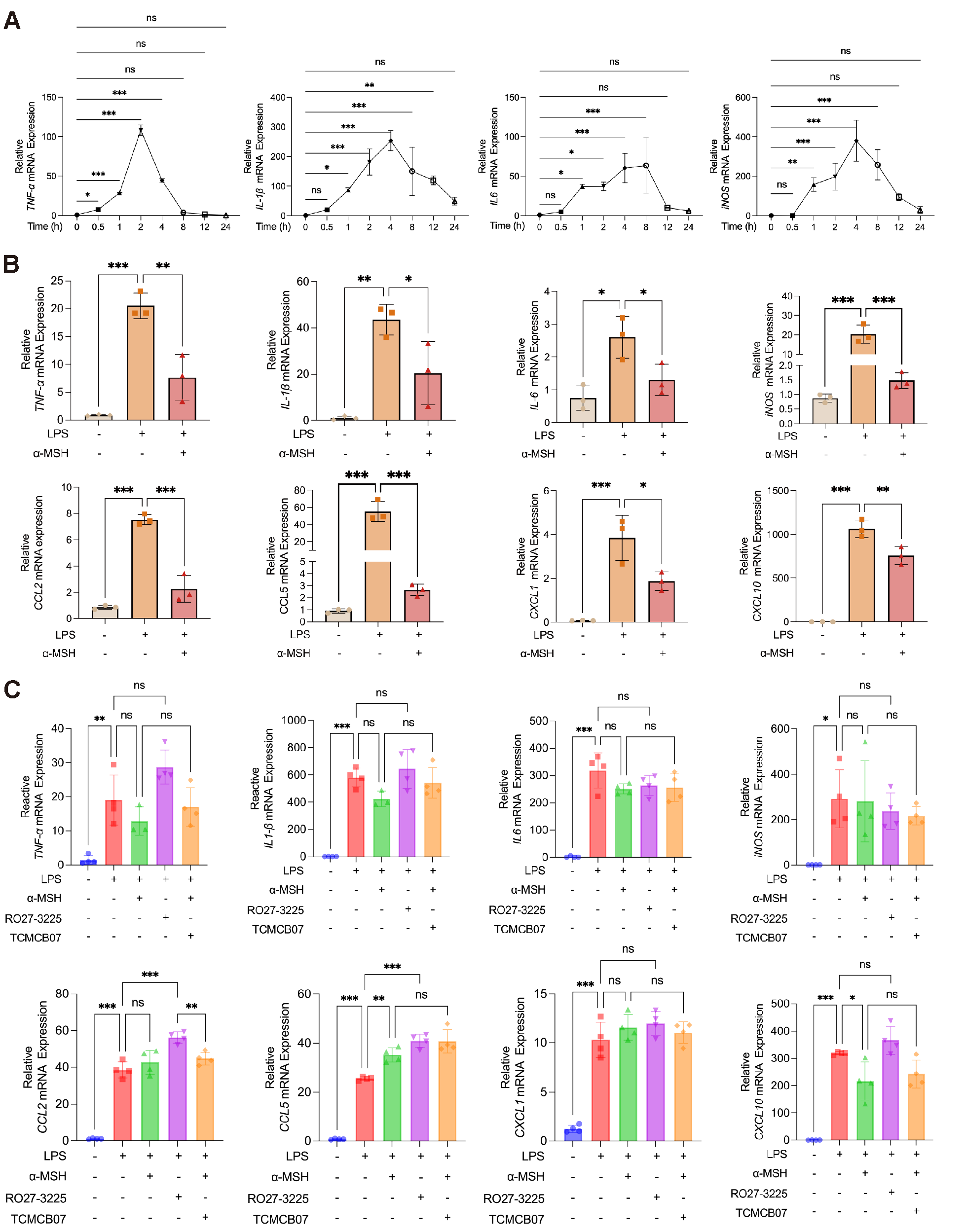


**Supplement Fig. 7. The effect of α-MSH on inflammatory-injury astrocytes.**

(A) The effects of LPS stimulation at different time points on inflammatory factor expression in astrocytes. n = 4. (B) qPCR analysis of the mRNA expression of *TNF-α*, *IL-1β*, *IL-6*, *iNOS*, *CCL2*, *CCL5*, *CXCL1*, *CXCL10* after the treatment of LPS for 1 h. n = 3. (C) qPCR analysis of the mRNA expression of *TNF-α*, *IL-1β*, *IL-6*, *iNOS*, *CCL2*, *CCL5*, *CXCL1*, *CXCL10* after the treatment of LPS for 4 h. n = 4. One-way ANOVA is used in A, B.


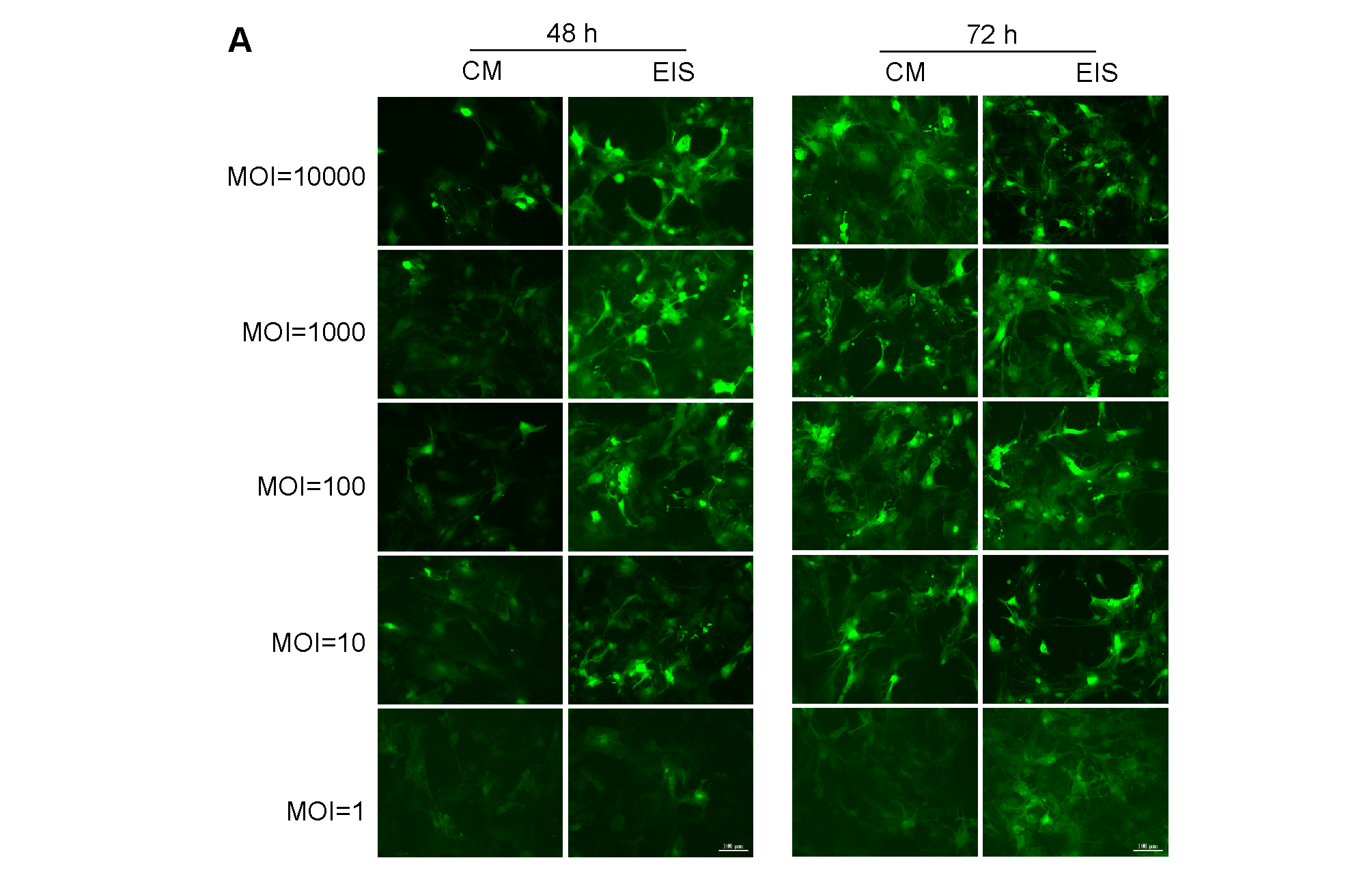


**Supplement Fig. 8.** **Efficiency of Ad-MC4R transfection into primary astrocytes.**

(A) Representative images of transfection with Ad-MC4R at different MOI at different time points. Scale bars: 100 μm.

**Supplement Tables**

**Supplement Table 1: The sequences of the primers.**

| primers | sequences |
| --- | --- |
| Mouse Icam-1-F | 5’-GTGATGCTCAGGTATCCATCCA-3’ |
| Mouse Icam-1-R | 5’-CACAGTTCTCAAAGCACAGCG-3’ |
| Mouse Vcam-1-F | 5’-AGTTGGGGATTCGGTTGTTCT-3’ |
| Mouse Vcam-1-R | 5’-CCCCTCATTCCTTACCACCC-3’ |
| Mouse P-selectin-F | 5’-GAAAGGGCTGATTGTGACCCC-3’ |
| Mouse P-selectin-R | 5’-AGTAGTTCCGCACTGGGTACA-3’ |
| Mouse E-selectin-F | 5’-ATGCCTCGCGCTTTCTCTC-3’ |
| Mouse E-selectin-R | 5’-GTAGTCCCGCTGACAGTATGC-3’ |
| Mouse TNF-α-F | 5’-CCCTCACACTCAGATCATCTTCT-3’ |
| Mouse TNF-α-R | 5’-GCTACGACGTGGGCTACAG-3’ |
| Mouse IL-1β-F | 5’-GCAACTGTTCCTGAACTCAACT-3’ |
| Mouse IL-1β-R | 5’-ATCTTTTGGGGTCCGTCAACT-3’ |
| Mouse IL-6-F | 5’-CCAAGAGGTGAGTGCTTCCC-3’ |
| Mouse IL-6-R | 5’-CTGTTGTTCAGACTCTCTCCCT-3’ |
| Mouse iNOS-F | 5’-GTTCTCAGCCCAACAATACAAGA-3’ |
| Mouse iNOS -R | 5’-GTGGACGGGTCGATGTCAC-3’ |
| Mouse CCL2-F | 5’-TTAAAAACCTGGATCGGAACCAA-3’ |
| Mouse CCL2-R | 5’-GCATTAGCTTCAGATTTACGGGT-3’ |
| Mouse CCL5-F | 5’-GCTGCTTTGCCTACCTCTCC-3’ |
| Mouse CCL5-R | 5’-TCGAGTGACAAACACGACTGC-3’ |
| Mouse CCL20-F | 5’-GCCTCTCGTACATACAGACGC-3’ |
| Mouse CCL20-R | 5’-CCAGTTCTGCTTTGGATCAGC-3’ |
| Mouse CXCL1-F | 5’-CTGGGATTCACCTCAAGAACATC-3’ |
| Mouse CXCL1-R | 5’-CAGGGTCAAGGCAAGCCTC-3’ |
| Mouse CXCL2-F | 5’-CAGACTCCAGCCACACTTCA-3’ |
| Mouse CXCL2-R | 5’-AGGTACGATCCAGGCTTCCC-3’ |
| Mouse CXCL10-F | 5’-CCAAGTGCTGCCGTCATTTTC-3’ |
| Mouse CXCL10-R | 5’-GGCTCGCAGGGATGATTTCAA-3’ |
| Mouse MC1R-F | 5’-CAACCTCATTGACGTGCTCAT-3’ |
| Mouse MC1R-R | 5’-TAACGCAGCGCATAGAAGATG-3’ |
| Mouse MC2R-F | 5’-ACACCGCAAGAAATAACTCCG-3’ |
| Mouse MC2R-R | 5’-AGGAGGACAATCAAGTTCTCCA-3’ |
| Mouse MC3R-F | 5’-TCCGATGCTGCCTAACCTCT-3’ |
| Mouse MC3R-R | 5’-GGATGTTTTCCATCAGACTGACG-3’ |
| Mouse MC4R-F | 5’-TGCTCGCATCCATTTGCAG-3’ |
| Mouse MC4R-R | 5’-ATGATCCCGACCCGCCTAA-3’ |
| Mouse MC5R-F | 5’-AGCCCGGTAAACAGAAGATTCA-3’ |
| Mouse MC5R-R | 5’-CTCTGAGGCGTTCAGGGTAAG-3’ |
| Mouse GAPDH-F | 5’-AGGTCGGTGTGAACGGATTTG-3’ |
| Mouse GAPDH-R | 5’-TGTAGACCATGTAGTTGAGGTCA-3’ |

**Supplement Table 2: Antibodies for immunofluorescence staining.**

| Antibodies | Dilution ratio | Article number | | Company |
| --- | --- | --- | --- | --- |
| Rat-anti-mouse CD31 | 1:200 | | ab7388 | Abcam |
| Rabbit-anti-mouse albumin | 1:300 | | ab207327 | Abcam |
| Rabbit-anti-mouse claudin5 | 1:200 | | ab131259 | Abcam |
| Rabbit-anti-mouse occludin | 1:200 | | 40-4700 | Invitrogen |
| Rabbit-anti-mouse GFP | 1:2000 | | HY-P80141 | MCE |
| Goat-anti-mouse GFAP | 1:200 | | ab53554 | Abcam |
| Rabbit-anti-mouse MC4R | 1:200 | | DF4984 | Affinity Biosciences |
| Goat-anti-mouse AIF-1/IBA1 | 1:100 | | NB100-1028 | Novus |
| Chicken-anti-mouse MAP2 | 1:200 | | ab5392 | Abcam |
| Donkey anti Rat IgG (H+L) (Alexa Fluor® 488) | 1:500 | | A-21208 | Invitrogen |
| Donkey anti Goat IgG (H+L) (Alexa Fluor® 488) | 1:500 | | A-11055 | Invitrogen |
| Donkey anti Rabbit IgG (H+L) (Alexa Fluor® 555) | 1:500 | | A-31572 | Invitrogen |
| Donkey anti Goat IgG (H+L) (Alexa Fluor® 555) | 1:500 | | A-21432 | Invitrogen |
| Donkey anti Chicken IgG (H+L) (Alexa Fluor® 555) | 1:500 | | A-78949 | Invitrogen |

**Supplement Table 3: Antibodies for FCM.**

| Antibodies | Dilution ratio | Article number | | Company |
| --- | --- | --- | --- | --- |
| Percp/cyanine5.5 anti-mouse-CD4 | 1:100 | | 100434 | BioLegend |
| PE/Cyanine7 anti-mouse-IFN-γ | 1:50 | | 505826 | BioLegend |
| APC anti-mouse-IL-17A | 1:50 | | 506916 | BioLegend |
| PE/Cyanine7 anti-mouse-CD45 | 1:100 | | 103114 | BioLegend |
| APC anti-mouse-CD11b | 1:100 | | 101212 | BioLegend |
| Percp/cyanine5.5 anti-mouse-Ly6C | 1:100 | | 128012 | BioLegend |
| PE anti-mouse-Ly6G | 1:100 | | 127607 | BioLegend |

**Supplement Table 4: Antibodies for WB.**

| Antibodies | Dilution ratio | Article number | | Company |
| --- | --- | --- | --- | --- |
| Rabbit-anti-mouse α-MSH | 1:500 | | Orb13589 | Biorbyt |
| Rabbit-anti-mouse MC4R | 1:500 | | DF4984 | Affinity Biosciences |
| Rabbit-anti-mouse p-p38 | 1:1000 | | 4511T | CST |
| Rabbit-anti-mouse p38 | 1:800 | | AF6456 | Affinity Biosciences |
| Rabbit-anti-mouse p-ERK | 1:800 | | AF1015 | Affinity Biosciences |
| Rabbit-anti-mouse ERK | 1:800 | | AF0155 | Affinity Biosciences |
| Rabbit-anti-mouse p-JNK | 1:800 | | AF3318 | Affinity Biosciences |
| Rabbit-anti-mouse JNK | 1:800 | | AF6318 | Affinity Biosciences |
| Rabbit-anti-mouse p-p65 | 1:800 | | AF2006 | Affinity Biosciences |
| Rabbit-anti-mouse p65 | 1:800 | | AF5006 | Affinity Biosciences |
| Rabbit-anti-mouse p-IκBα | 1:800 | | AF2002 | Affinity Biosciences |
| Rabbit-anti-mouse IκBα | 1:800 | | AF5002 | Affinity Biosciences |
| Mouse-anti-mouse GAPDH | 1:2000 | | 2B5 | Abbkine |
| Mouse-anti-mouse β-Tubulin | 1:2000 | | 3G6 | Abbkine |
| Horse radish peroxidase labeled goat anti-mouse IgG (H+L) | 1:5000 | | ΑΒ-2305 | ZSGB-BIO |
| Horse radish peroxidase labeled goat anti-rabbit IgG (H+L) | 1:5000 | | ΑΒ-2301 | ZSGB-BIO |

**Supplement Table 5: Reagents for in vivo and in vitro experiments.**

| Reagents | Article number | Company |
| --- | --- | --- |
| RO27-3225 | HY-P2242A | MCE |
| TCMCB07 | HY-P5971 | MCE |
| α-MSH | HY-P0252 | MCE |
| U0126 | SJ-MX0205A | SparkJade |
| SP600125 | SJ-MX0185 | SparkJade |
| SB203580 | SJ-MX0092 | SparkJade |
| NF-κB-IN-11 | HY-155998 | MCE |
