# Supplementary material for: Electroacupuncture alleviates blood-brain barrier disruption and neuroinflammation via astrocytic MC4R in a mouse model of multiple sclerosis
Source: J Neuroinflammation. 2025 Dec 26;23:40. doi: 10.1186/s12974-025-03667-1 (PMC12849424; doi:10.1186/s12974-025-03667-1)
Supplement: Supplementary file 4 — Supplementary Material 4. [file 12974_2025_3667_MOESM4_ESM.docx]

**Supplementary uncropped Gels and Blots images**


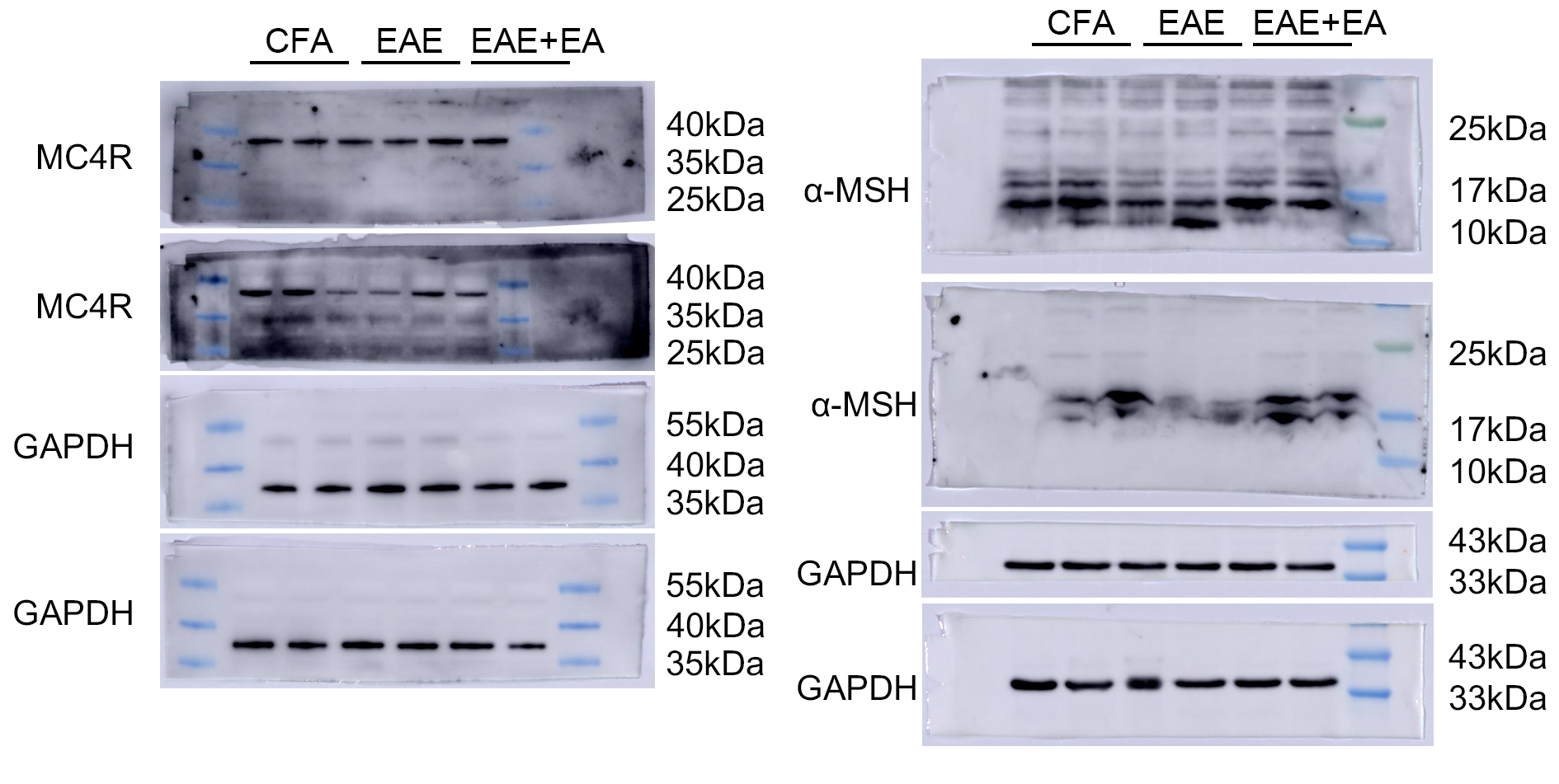


The full uncropped Gels and Blots images of Fig. 1F, G, H, I.


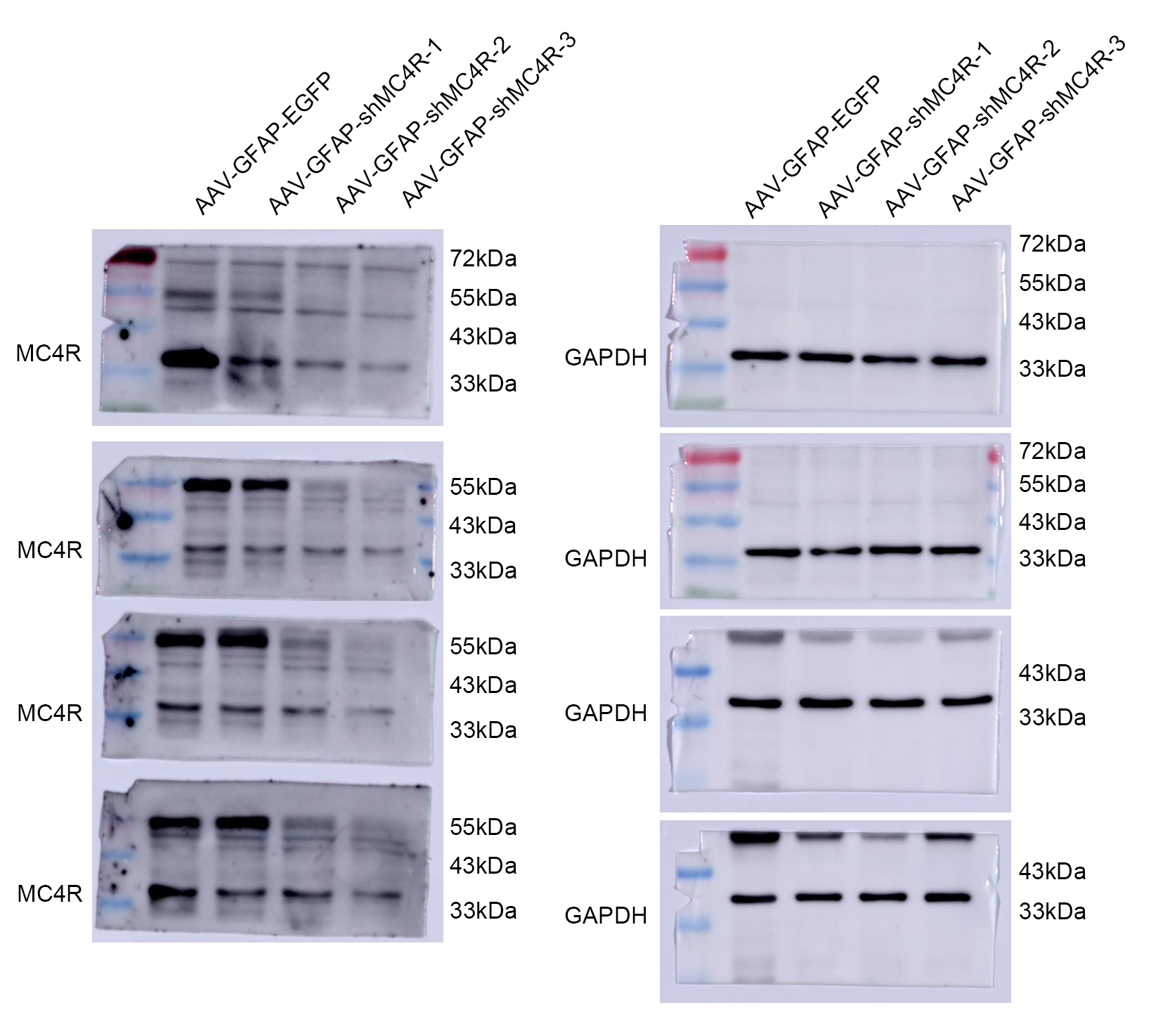


The full uncropped Gels and Blots images of Fig. 4C, D.


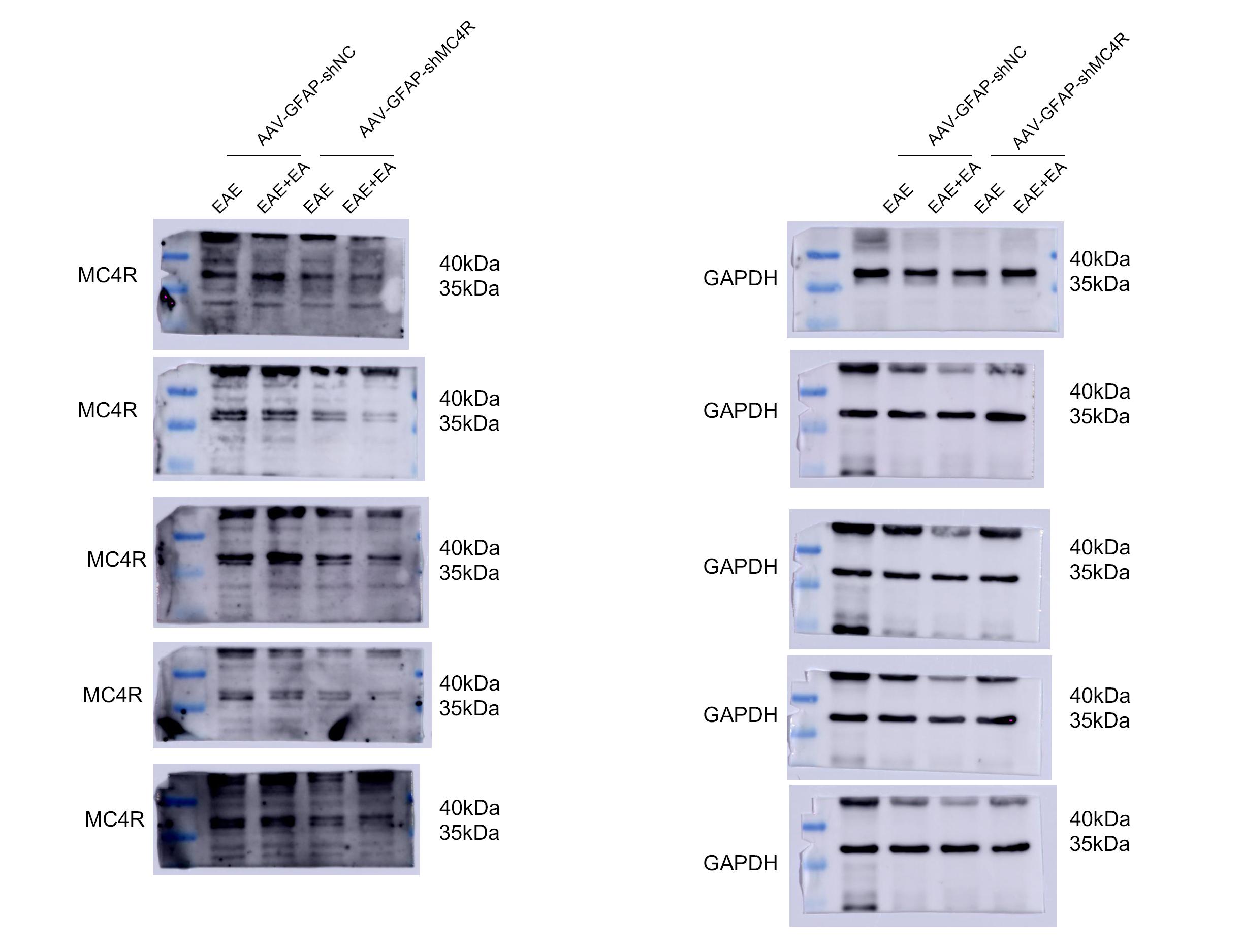


The full uncropped Gels and Blots images of Fig. 6F, G.


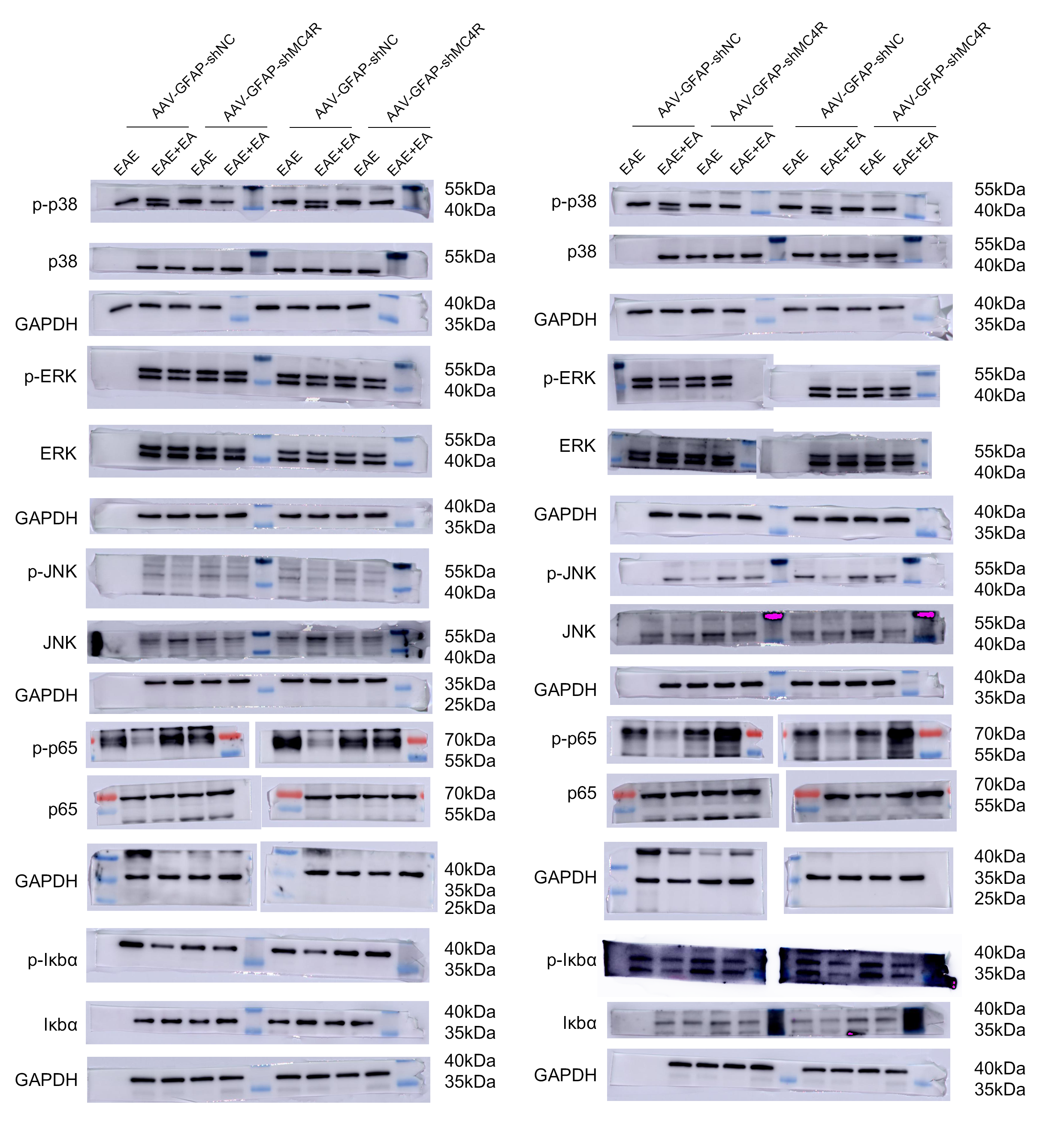


The full uncropped Gels and Blots images of Fig. 6F, H-L.


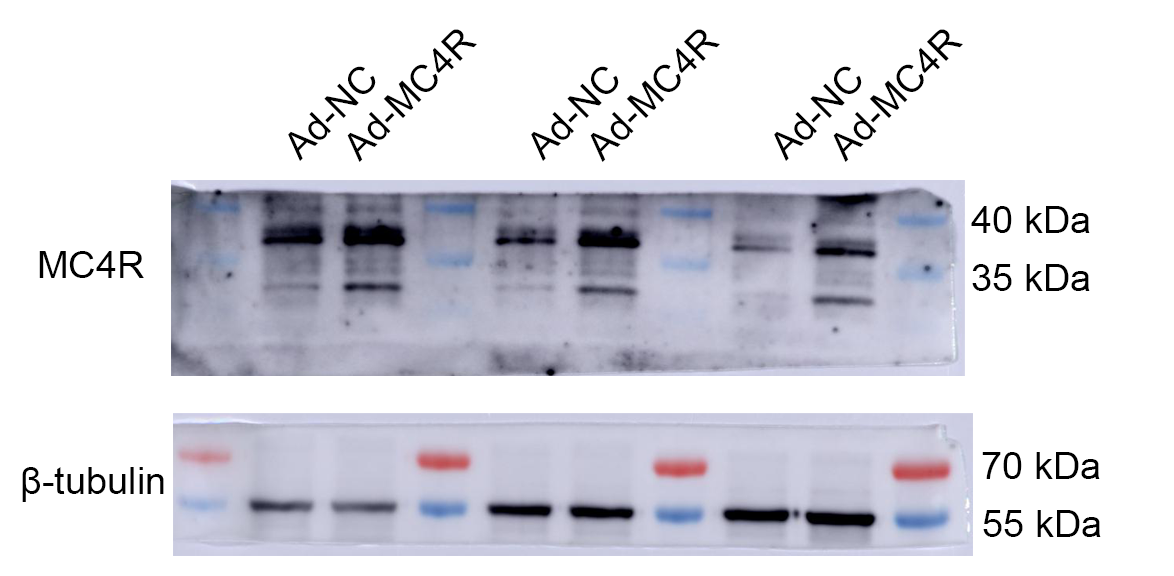


The full uncropped Gels and Blots images of Fig. 7B, C.


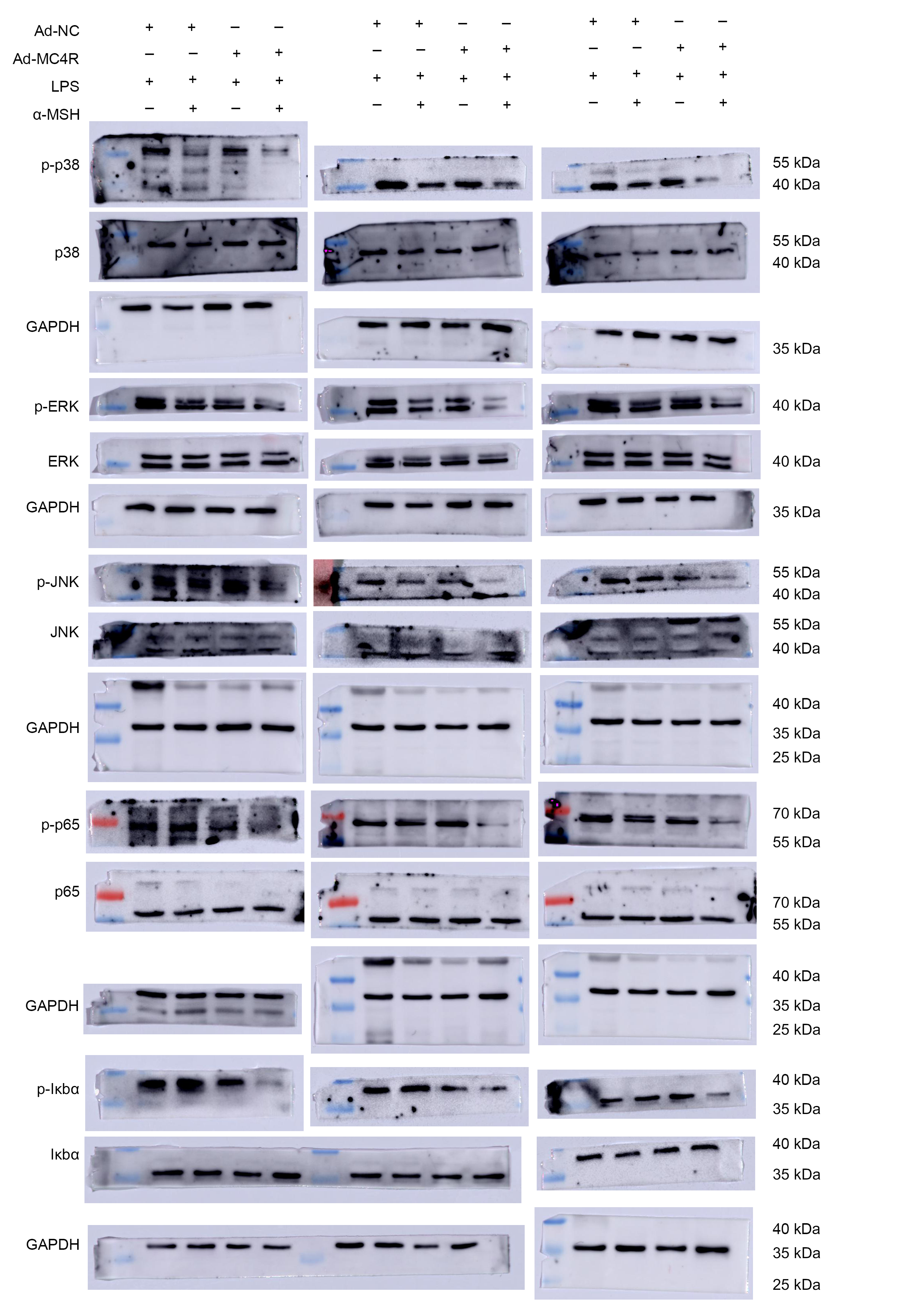


The full uncropped Gels and Blots images of Fig. 7L, M.


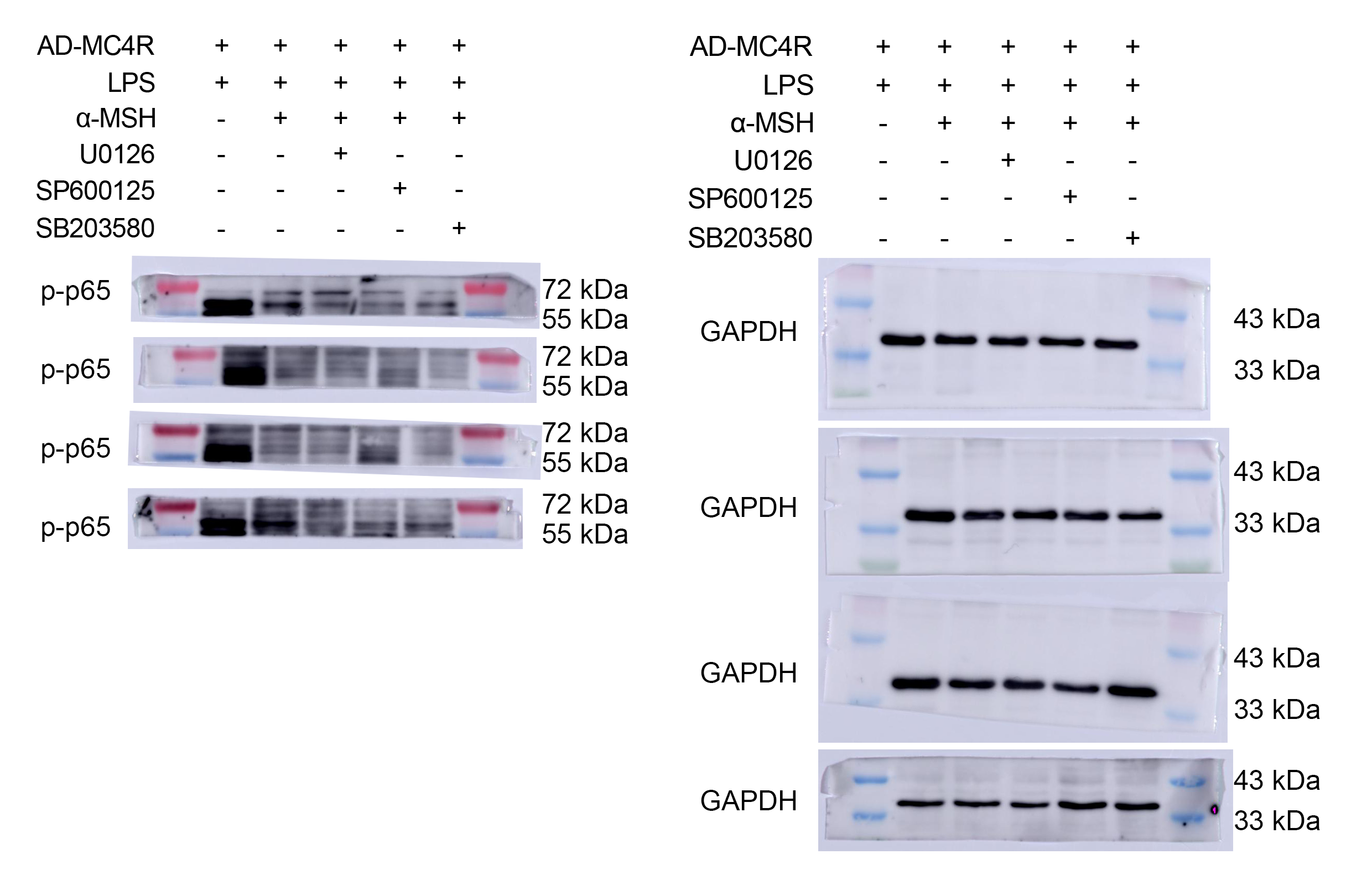


The full uncropped Gels and Blots images of Fig. 8B.
